# Morphological characterization, pathogenicity screening, and molecular identification of *Fusarium* spp. isolates causing post-flowering stalk rot in maize

**DOI:** 10.3389/fmicb.2023.1121781

**Published:** 2023-03-31

**Authors:** J. Harish, Prashant P. Jambhulkar, Ruchira Bajpai, Meenakshi Arya, Piyoosh K. Babele, Sushil K. Chaturvedi, Anil Kumar, Dilip K. Lakshman

**Affiliations:** ^1^Department of Plant Pathology, College of Agriculture, Rani Lakshmi Bai Central Agricultural University, Jhansi, Uttar Pradesh, India; ^2^College of Agriculture, Rani Lakshmi Bai Central Agricultural University, Jhansi, Uttar Pradesh, India; ^3^USDA-ARS, Beltsville, MD, United States

**Keywords:** maize, *Fusarium*, PFSR, morphology, pathogenicity, soilborne pathogens, molecular characterization, translation elongation factor 1 α

## Abstract

Post flowering stalk rot (PFSR) of maize caused by the *Fusarium* species complex is a serious threat to maize production worldwide. The identification of *Fusarium* species causing PFSR based on morphology traditionally relies on a small set of phenomic characteristics with only minor morphological variations among distinct *Fusarium* species. Seventy-one isolates were collected from 40 sites in five agro-climatic zones of India to assess the diversity of *Fusarium* spp. associated with maize crops showing symptoms of PFSR in the field. To investigate the pathogenicity of *Fusarium* spp. causing PFSR sixty isolates were toothpick inoculated between the first and second node at 55 days after sowing during the tassel formation stage of the crop in Kharif (Rainy season), and *Rabi* (Winter season) season field trials. Ten most virulent Fusarium isolates, based on the highest observed disease index, were identified by homology and phylogenetic analyses of partial sequences of the translation elongation factor 1 α (Tef-1α). Based on morphological traits such as mycelial growth patterns and *mycelial* pigmentation, *Fusarium* isolates were divided into nine clusters. The isolates were judged to be virulent based on their ability to decrease seedling vigour in *in-vivo* situations and high disease severity in field experiments. Pathogenicity test during the *Kharif* season showed 12 isolates with virulent disease symptoms with a mean severity ranging between 50 to 67 percent disease index (PDI) whereas in *Rabi* season, only five isolates were considered virulent, and the mean severity ranged between 52 to 67 PDI. Based on pathological characterization and molecular identification, 10 strains of *Fusarium* species namely, *Fusarium acutatum* (2/10), *Fusarium verticillioides* (Syn. *Gibberella fujikuroi* var. *moniliformis*) (7/10), *Fusarium andiyazi* (2/10) recorded the highest diseases index. All these species are part of the *Fusarium fujikuroi* species complex (FFSC). The distribution of virulent isolates is specific to a geographical location with a hot humid climate. Increased knowledge regarding the variability of *Fusarium* spp. responsible for PFSR of maize occurring across wide geographical locations of India will enable more informed decisions to be made to support the management of the disease, including screening for resistance in maize-inbred lines.

## Introduction

Maize (*Zea mays* L.) is the third most cultivated cereal grain in the world and contributes greatly to global food security. Maize can be grown in temperate tropical and subtropical climates (Souki et al., 2011). With the development of agriculture, infectious plant diseases have become an increasingly significant factor affecting crop yield and economic efficiency. Maize crop is often infected by several plant pathogens (bacteria, viruses, fungi, nematodes, etc.) that are detrimental to the yield and quality of grains and eventually threaten food security around the globe (Tiru et al., 2021). Pathogenic diseases of maize are one of the major obstacles leading to hefty economic, nutritional, and livelihood impacts. Among several pathogenic diseases, fungal diseases caused by *Fusarium* spp. are most threatening to the yield and quality of grains. Moreover, fungal infection and consequent contamination with mycotoxins pose serious human and animal health hazards, following ingestion of contaminated food and feeds. Post flowering stalk rot (PFSR) of maize is a serious diseases affecting maize production worldwide. PFSR is mainly caused by the *Fusarium fujikuroi* species complex (FFSC) which includes more than 60 *Fusarium* species (both phytopathogenic and clinical importance) (Yilmaz et al., 2021). The pathogens of FFSC responsible for causing PFSR are *Fusarium verticillioides* (Sacc.) Nirenberg, *Fusarium subglutinans* (Wollenw. and Reinking) P.E. Nelson, Toussoun and Marasas, *Fusarium graminearum* Schwabe, *Fusarium proliferatum* (Matsush.) Nirenberg ex Gerlach and Nirenberg, and *Fusarium oxysporum* Schltdl (Gherbawy et al., 2002; Harleen et al., 2016). This complex is a collective group of fungi that lives in the soil and spreads through plant roots and crowns. PFSR-infected maize plants exhibit symptoms of drooping, wilting, and drying of leaves, empty cob development, and an increase in the angle between stalks and cobs in the field. *Fusarium verticillioides* has been reported as a major fungal species of PFSR (Venturini et al., 2013; Yu et al., 2017; Jambhulkar et al., 2022). The isolates of *Fusarium* spp. from various agroclimatic regions exhibit significant variability in their pathogenicity, genetics, reproductive, and toxicity characteristics (Danielsen et al., 1998; Chandra Nayaka et al., 2008; Venturini et al., 2013; Qiu et al., 2015). The pathogenic potential and cultural variation of these continuously evolving plant pathogens depend on geographical locations, season/weather, and cropping patterns (Kavas et al., 2022).

The majority of maize-growing states in India, especially rainfed regions, such as Jammu and Kashmir, Punjab, Haryana, Delhi, Rajasthan, Madhya Pradesh, Uttar Pradesh, Bihar, West Bengal, Andhra Pradesh, Tamil Nadu, and Karnataka are prone to PFSR infection (Kaur and Mohan, 2012). During the *Kharif* season (Rainy season), *Macrophomina phaseolina* (Tassi) Goid is the principal pathogen responsible for PFSR in India, which develops when there is a protracted dry spell. In other parts of the country where maize is grown under guaranteed irrigation, *F. verticillioides* is primarily responsible for PFSR. However, several other *Fusarium* spp. are also associated with this disease, which affects approximately 27.0 to 77% of maize plants (Mamatha et al., 2020). All India Co-ordinated Research Project on Maize (AICRP-Maize) 2014, reported that this disease causes 22 to 64% yield losses. In different agroecological zones of India, multiple *Fusarium* spp. exist with varying pathogenic potential, making PFSR control strategies very challenging and often unsuccessful. The virulence pattern of *Fusarium* spp. isolates in not unclear. Moreover, being a soilborne pathogen, management of the disease through a fungicide application is difficult, costly, and destructive to ecology (Rahjoo et al., 2008; Harleen et al., 2016). Therefore, morphological characterization and pathogenicity screening of *Fusarium* spp. causing PFSR is critical for preliminary identification and classification and eventually for developing efficient disease management strategies.

Several prior studies showed variability in cultural and microscopic characteristics of several *Fusarium* spp. isolated from Rajasthan (Khokhar et al., 2014), Punjab (Harleen et al., 2016), Karnataka (Ramesha and Naik, 2017), and Telangana (Mamatha et al., 2020) states of India. These reports indicate region-specific variability in several *Fusarium* spp. of these states. We argue that a more comprehensive and comparative investigation of these PFSR-causing pathogens across wide geographical locations of India is necessary to delineate the virulence behaviour of these isolates and to design their management strategies. Moreover, being a soilborne pathogen, management of the disease through conventional measures is difficult such as fungicide application which is cost-prohibitive and destructive to ecology. A substantial amount of work has been done to investigate the use of biological control to address the issue of PFSR (Chandra Nayaka et al., 2010; Wu et al., 2015; Lu et al., 2020; Jambhulkar et al., 2022), but breeding resistant germplasm is the ultimate solution to reduce crop loss due to PFSR. Therefore, stable resistant inbred lines against virulent isolates of *Fusarium* spp. will be useful as resistant sources against PFSR.

This study aims to comprehensively characterize morphologies of *Fusarium* spp., isolated from five agro-climatically distinct states *viz*. Rajasthan, Gujarat, Madhya Pradesh, Telangana, Karnataka, and Andhra Pradesh of India. The respective *Fusarium* isolates were cultured from rotten maize stems exhibiting signs of PFSR, such as drooping cobs and desiccated plants. Moreover, we examined the pathogenicity of the collected isolates to identify the most virulent ones. Increased knowledge regarding the variability of *Fusarium* spp. responsible for PFSR of maize occurring across wide geographical locations of India will enable more informed decisions to be made to support the management of the disease.

## Materials and methods

### Sampling and fungal strain isolation

**FIGURE 1:**
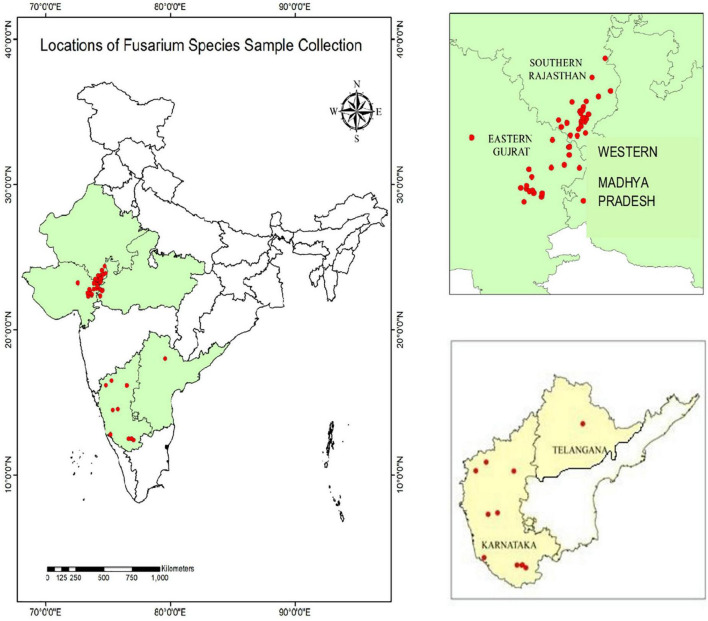
Geographical locations indicating sampling sites of *Fusarium* spp. Isolates.

During 2020 and 2021, maize plants infected by PFSR were randomly gathered from 40 sites in southern Rajasthan, eastern Gujarat, western Madhya Pradesh, Karnataka, and Telangana. A few locations of sample collection performed during Rabi 2020 are Banswara, Survaniya, Bodla, Karji, Hamirpura, Rakho, Badodiya Kali Pahari, Kalinjara, Sagdod, Devliya, Kushalgarh, Sajjangarh, Monadungar villages Ghatol, Senavasa, Badgaon, Chanduji ka Gada in Southern Rajasthan. Eastern Gujarat: Dahod, Godhra, Pavagarh, Vadodara. In Karnataka, samples were taken from Mandya, Haveri, Davanagere, Mysore, Raichur, Belagavi, and Bagalkot, among other locations. One isolate was recovered from Warangal in Telangana. Geographical locations depicting the latitude and longitude of *Fusarium* spp. sample collection is given in Supplementary Table 1. These locations are part of five agro-climatic zones *viz*. Eastern plateau and hills, Central Plateau and hills, Western plateau and hills, Southern plateau and hills, and Gujarat plain and hills. On the India map, each collection site’s latitude and longitudinal position (GPS data) were recorded and mapped (Figure 1).

These sample collection locations are part of five agro-climatic zones in India *viz*. Eastern plateau and hills, Central Plateau and hills, Western plateau and hills, Southern plateau and hills, and Gujarat plain and hills. To isolate the pathogens, all adhering soil particles were removed from the vascular pith tissues of infected maize plants by washing them with tap water. The pith of the infected stalk was surface sterilized by soaking in 0.1% sodium hypochlorite for 30 s before being rinsed twice in sterile distilled water, dried on sterile blotting paper, and placed on potato dextrose agar plates supplemented with streptomycin sulphate (100 μg ml^–1^) to prevent bacterial contamination and incubated in the dark. The isolates were moved from potato dextrose agar (PDA) to Spezieller Nahrstof farmer agar (SNA) medium (1 g KH_2_PO4, 1 g KNO_3_, 0.5 g MgSO_4_.7H_2_O, 0.5 g KCl, 0.2 g glucose, 0.2 g sucrose, and 20 g agar in 1 L SDW), to enhance sporulation, according to Leslie and Summerell (2006). Seventy-one isolates of *Fusarium* pathogen cultures from infected stalks were purified by isolating a single spore. The isolates were sub-cultured on potato dextrose agar slants and allowed to grow at 27 ± 1°C in the dark for 15 days. These slants were stored in a refrigerator at 4°C and utilized throughout the study.

### Cultural and morphological characterization of isolates

To investigate the morphology of various *Fusarium* isolates, isolates were individually cultured on PDA. A 5 mm culture disc was cut from the edge of the actively developing culture plates and positioned at the centre of fresh PDA plates and incubated in the dark at 27 ± 1°C. After 7–8 days, observations of culture phenotypes were recorded considering colony diameter, colour, margins, and general appearance from all the Petri dishes. Each isolate was maintained in triplicate. Thereafter, micro-morphological characterization was conducted by observing fungal cells under a microscope. Eventually, to study conidial morphology, culture explants were transferred and grown onto SNA Petri dishes. Slides were prepared from four-day-old fungal cultures on SNA medium to promote the sporulation and production of both microconidia and macroconidia. The length, width, and number of septations per conidia were recorded and all the microphotographs were captured using a CILIKA^®^ digital microscope (Medprime, Thane, Mumbai, India) at 40X magnification.

### *In-vitro* pathogenicity test

The germination paper/paper towel method (Flint-Garcia et al., 2005) was used to determine the pathogenicity of specific *Fusarium* isolates *in vitro*. A total of 71 *Fusarium* isolates were examined on PC-4 maize seeds. Conidial suspensions of 2x10^7^cfu/ml were made by scraping mycelium into sterile distilled water in a test tube. Surface-sterilized maize seeds were soaked for 30 min in test tubes containing a suspension of each inoculum. After pathogen treatment, 10 maize seeds per inoculum are placed in moist germination paper, labelled, rolled, and placed in a seed germinator with controlled conditions (28°C temperature and 80% relative humidity). The initial count for seed germination, shoot, and root length, was recorded on the eighth day, followed by the tenth day, and the final count on the twelfth day, respectively. Pathogenicity of the *Fusarium* isolates and their influence on seed germination, viability, and vitality were recorded. The Dezfuli et al. (2008) formula was utilized to calculate seedling vigour.


V⁢i⁢g⁢o⁢u⁢r⁢i⁢n⁢d⁢e⁢x⁢V⁢I=Germination(%)×Totalseedlinglength(cm)

### Pathogenicity studies during *Kharif* and *Rabi* seasons in field condition

The field experiments for pathogenicity evaluation were conducted in the research farm of Rani Lakshmi Bai Central Agricultural University, Jhansi (N 25°30′55.75′′ E 78°32′47.31′′), India. Seeds of a cultivated variety with intermediate susceptibility “Pusa Composite 4” were seeded in the field adopting a Randomized Block Design with three replications of each isolate during *Kharif* (rainy season) 2020 and *Rabi* (winter season) 2020-21. Standard agronomic practices adhered to for the soil preparation and fertilizer application. The spacing between plants was 30 cm, while the spacing between rows was maintained at 60 cm.

### Toothpick inoculation method

Round wooden 6 cm-long toothpicks were sterilized by boiling in distilled water for 1 h. Any impurities such as gum, resin, or any other particles that may limit fungus growth were discarded by decanting the water, and the process was repeated three times. Following sterilization, the toothpicks were dried on sterile blotting paper. Ten toothpicks were put into each test tube, and PDB (Potato dextrose broth; Hi-Media^®^) was added to each tube to thoroughly moisten the toothpicks. The pointed tips of the toothpicks were maintained in an upward position and the volume of broth in a test tube were such that the 1–1.5 cm tip of the toothpicks remained above the broth level. The test tubes were autoclaved, cooled, and then infected with *Fusarium* isolate plugs. The inoculated test tubes were incubated at 27 ± 1 °C with 12 h of alternating light and darkness for 10 days until the maximal mycelial growth took place on toothpick tips. Sixty isolates [FUR14, FUG16, FUR13, F38, F27, F3, F2, F43, FUG7, F19, FUG4, F9, F55, F58, F44, F16, F47, FUG1, F45, FUG2, F10, F7, F6, F31, F36, F28, FUG5, F34, F8, F22 (Dungarpur), F46, FUG6, F12, F33, F14, F26, F48, F20, F4, F11, F49, FUG8, F42, D2, FUG3, F52, F57, FUR10, F21, F18, FUR15, F13, F1, F25, FUR12, F59, Chokhla, F32, FUR11, and F35] were used in pathogenicity test during *Kharif* season 2020 and the nine isolates FUR14, FUG16, FUR13, F38, F27, F3, F2, F43, FUG7 which showed less virulence during *Kharif* season 2020, were excluded for the subsequent trial. For the pathogenicity trial in the *Rabi* season of 2020-21, the 51 *Fusarium* isolates from the 2020 *Kharif* season and nine new isolates collected from the southern region of India, *viz-a-viz*, Mandya, Mandya 2, Haveri, Mysore, W3-2, G1-3, B1-1, Davanagere, and Raichur were included in the study.

At the tasselling stage, 45 to 50-day-old maize plants were artificially inoculated. Five plants per replication were inoculated with each *Fusarium* isolate. The lower internodes, particularly the second internode above the soil’s surface in each plant, were inoculated with mycelium. The fungus-colonized toothpicks were inserted diagonally after puncturing the stem and creating a 2-centimetre hole in the first internodes with a jabber. The onset of symptoms initiated 20 to 25 days following vaccination. At the time of harvest, disease intensity and severity (PDI) were computed using Payak and Sharma (1983) 1 to 9 scale. Lesion length was measured by split opening the inoculated stalk at harvest to determine the percentage disease index (Chester, 1950; Chaube and Singh, 1991; Vieira et al., 2012).


P⁢e⁢r⁢c⁢e⁢n⁢t⁢d⁢i⁢s⁢e⁢a⁢s⁢e⁢i⁢n⁢d⁢e⁢x=

$$\frac{s\,u\,m\,o\,f\,n\,u\,m\,e\,r\,i\,c\,a\,l\,r\,a\,t\,i\,n\,g}{T\,o\,t\,a\,l\,n\,u\,m\,b\,e\,r\,o\,f\,s\,a\,m\,p\,l\,e\,t\,a\,k\,e\,n\times M\,a\,x\,i\,m\,u\,m\,g\,r\,a\,d\,e}\times 100$$

The disease rating scale to measure the disease severity of PFSR is given by Payak and Sharma (1983). (1) No discoloration or discoloration only at the point of inoculation; (2) less than 25% of the inoculated internode discoloured; (3) 25 to < 50 % of the inoculated internode discoloured; (4) 50 to < 100 % of the inoculated internode discoloured; (5) 25% of adjacent internode discoloured; (6) half discoloration of the adjacent internode; (7) Discolouration of three internodes; (8) Discolouration of four internodes, and (9) Discolouration of five internodes or plants prematurely killed.

### Isolation of genomic DNA

The genomic DNAs of isolates were extracted using the CTAB method (Li et al., 2013). The 10 most virulent *Fusarium* isolates were incubated on PDA plates at 27 ± 2°C under the dark for 6–7 days. An approximately 1 cm^2^ of fungal mycelium was transferred from culture plates to a sterile 2 ml Eppendorf tube. Mycelium was macerated using a tissue homogenizer and 500 μl CTAB buffer, followed by incubation of the Eppendorf tube at 60°C for 1 h in a hot water bath (Cole-Palmer India Pvt. Ltd. Mumbai) with a gentle shaking at 10 min interval. The composition of the buffer was (2.5% CTAB, 4 M NaCl, 20 mM EDTA, 100 mM Tris-HCl, 0.2% β- Mercaptoethanol; pH 8.0). All components for the buffer preparation were procured from (Hi-media, India). The concentration and purity of DNA were determined using Nanodrop (NanoDrop™ ThermoFisher™ Scientific, Mumbai, India) and recording its absorbance at (260/280 nm) and the quality of DNA through agarose gel electrophoresis (0.8%) (w/v) (iGene Labserve, Delhi, India). The gel was observed under the Gel documentation system (Syngene^®^InGenius3, Frederick, USA). The final volume of DNA was made to 50 ng.

### Phylogenetic analysis of the TEF-1α gene sequence

Sequences of the translation elongation factor 1α (TEF-1α) gene from each isolate were amplified using the Tef-1α EF-1 [5’-ATGGGTAAGGA (A/G) GACAEAGAC-3’] and EF-2 [5’- GGA (G/A) GTACCAGT (G/C) ATCATGTT-3’] primer pairs (O’Donnell et al., 1998). PCR amplification (50 μl reaction volume) was performed with an initial denaturation at 94 °C for 5 min followed by 35 cycles each at 94 °C for 1 min, annealing at 50 °C for 1 min, extension at 72 °C for 2 mins followed by a final extension at 72°C for 10 mins, in a Thermocycler (Veriti™, Applied Biosystem™, New Delhi, India). The Tef-1α gene sequences of the ten most virulent *Fusarium* spp. isolates were sequenced through the Sanger sequencing method at Medauxin™, Bangalore. The obtained sequences were subjected to nBLAST to molecular identify at species levels through homology with available nucleotide sequences from the NCBI fungal DNA database^1^. The homology-identified sequences were deposited in the GenBank database of the National Centre for Biotechnology Information (NCBI), and Accession numbers were obtained. The reference Tef-1α nucleotide sequences of the *Fusarium* spp. showing closest homologies in the nBLAST search were retrieved from the FUSARIOID-ID database (Crous et al., 2021) for molecular phylogenetic relationships (*Fusarium verticillioides* Reference species: MW401977 CBS 117.28 strain, MW402080 CBS 141.59 strain; *F. andiyazi* Reference species: MN533989 CBS 119856 strain, MN193854 NRRL 31727 strain; *F. acutatum* Reference species: MW402124 CBS 401.97 strain, MW402125 CBS 402.97 strain). *Alternaria burnsii* isolate Alt-MP6 (Sequence ID: ON993391) was used as an outgroup. The phylogenetic tree was constructed by using MEGA X software using 1,000 bootstrap values and beautified by using ITOL software (Letunic and Bork, 2007).

### Statistical analysis

Arc GIS 10.1 platform (ESRI Inc., United States) was used for preparing the map for the study area and sample location sites. For hierarchical cluster analysis, all measurements of morphological and microscopic characters (including pigmentation, pattern, and type of mycelium, colony colour, macro and microconidia size, shape, and septation), were averaged and given values. A cluster analysis was conducted, and Unweighted Pair Group Method with Arithmetic mean (UPGMA) based dendrogram constructed (Jaccard’s coefficient) by using the software PAST 4.03. For each trait, descriptive statistics were calculated. Analysis of variance (ANOVA) was done by verifying the Shapiro-Wilk tests by SAS 9.1 (SAS, Inc., North Carolina, USA) software at 5% probability. Pair-wise mean comparison of disease severity between isolates during *Kharif* and *Rabi* seasons was calculated according to Tukey’s HSD test at *p* < 0.05. Data on pathogenic variability of virulent isolates under *in-vitro*, *Kharif*, and *Rabi* seasons were analysed by analysis of variance (ANOVA) and compared differences between percent reduction in seedling vigour and disease severity by virulent isolates using R (Gentleman, 2008). Arc sine transformation was done for percent data to make residuals normal and then back-transformed for graphical presentation. The significance of mean difference within percent reduction in vigour and disease severity by virulent strains was tested by Student’s *t*-test in combination with Bonferroni correction at *P* = 0.05 level of probability. Box plots were created to depict the distributions of seed germination parameters and shoot, root, and vigour index throughout growth days and trials. The analysis was performed at 0.05 or 0.01 significance level as indicated. The correlation study was performed using Jamovi version 1.2.27 at 5% level of significance.

## Results

### Identification of isolated cultures from maize stalk rot samples

#### Morphological characterization

**FIGURE 2:**
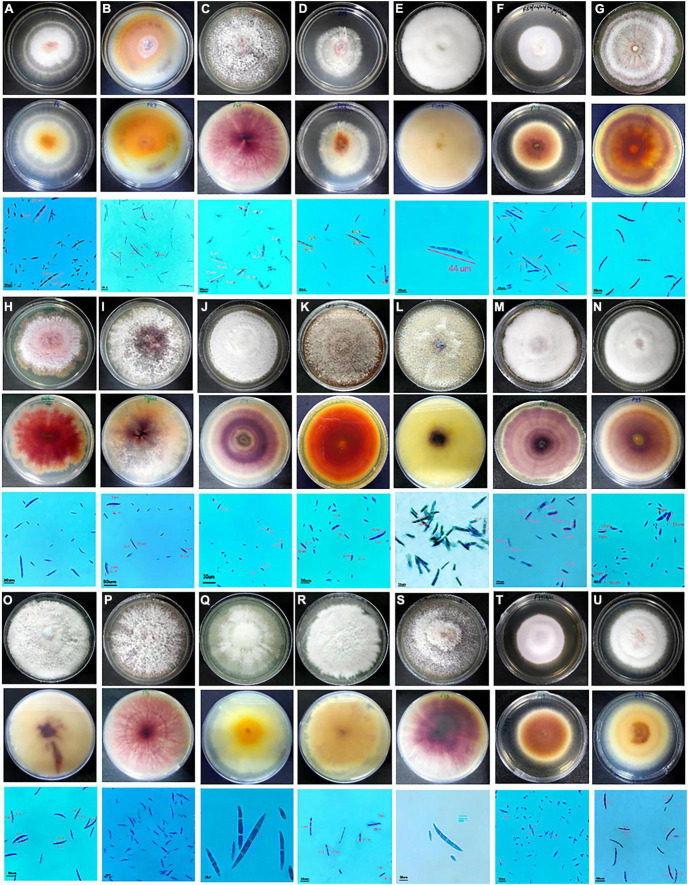
Cultural and morphological characteristics of *Fusarium* spp. causing PFSR in maize. For each isolate in the top row is the upper surface of the culture plate, the middle row is the lower surface, and the bottom row is a microscopic photograph of spores. **A**- F6; **B**- F27; **C**- F-47; **D**- F52; **E**, **F**- F59; **G**-F41; **H**- Raichur; **I**- Mysore; **J**- F19; K-F2; **L**- F13; **M**-F26; **N**-F33; **O**- F13; P-F14; **Q**- F8; **R**-F9; **S**- F58; **T**-F49; **U**-F28.

**FIGURE 3:**
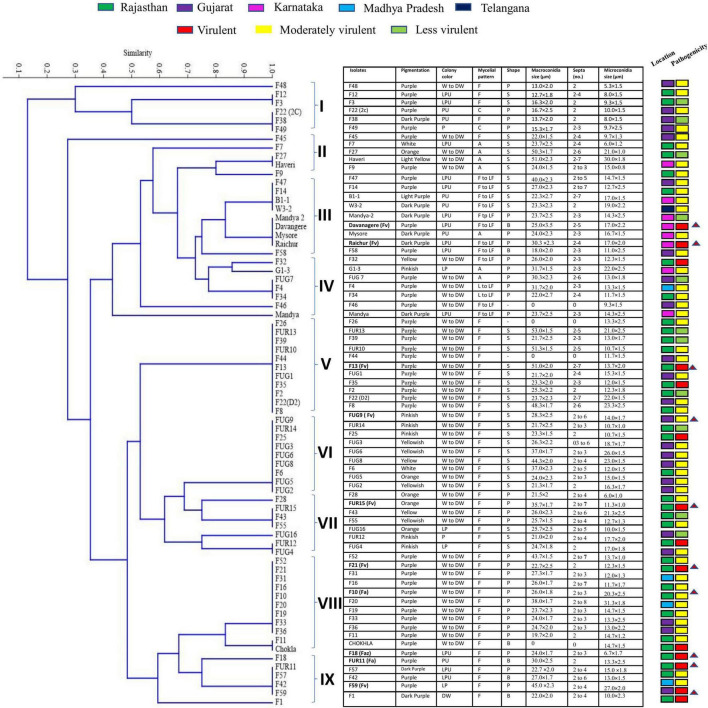
Dendrogram derived from morphological characters of 71 isolates of *Fusarium* spp. collected from 5 agroclimatic zones based on pigmentation, colony colour, mycelial pattern, shape and size of macro, and microconidia and number of septa in macroconidia grown on PDA medium.

*Fusarium* isolates display variability in several phenotypic characteristics such as colony colours, mycelium, pigmentation, sporulation, branching, conidial size, and shape. All the *Fusarium* isolates displayed a pronounced difference in their colony colours ranging in hue from violet, light violet, light pink, and light pink to light violet, dark violet, and filthy white. The colony colour of 47 of the *Fusarium* isolates was white to dirty white. The distribution of the pigments diffused in the culture agar of the 71 isolates were as follows: 42 purple, 7 dark purple, 6 pink, 8 yellow, and 1 light purple-2 isolates did not produce pigments. A significant variation in their colours and conidia among different isolates is depicted in Figure 2. The mycelial development of the *Fusarium* was considerably more variable. Some isolates exhibited white mycelial growth that was fluffy, white mycelial growth that was sparse, and white and purple mycelial growth that was mixed (Figure 2). All 71 isolates displayed a significant degree of variation in their mycelial size, colour, pigmentation, shape and size of macro and microconidia, and their septation. Among them, 51 isolates displayed fluffy mycelial development, 13 isolates were less fluffy, and 7 isolates were appressed. Among all 71 isolates, 30 had pointed macroconidia, 6 blunt-end conidia, and 35 sickle-shaped conidia. *Fusarium* isolates were divided into distinct clusters based on pigmentation, colony colour, mycelium pattern, mycelium type, conidial size, and shape. Based on morphological traits, 71 isolates were grouped into nine clusters (Figure 3). Cluster I consisted of six isolates with purple to dark purple pigmentation, purple to light purple, and a fluffy, condensed mycelium. Pointed to sickle-shaped macroconidia size was in the range of 13–15.3 μm with 2 septa. Microconidial size was in the range of 5.3–10.0 μm. Cluster II comprised of five isolates with purple, white, orange, and light-yellow pigmentation, white to dirty white colony colour, and fluffy to appressed mycelium. The size of sickle-shaped macroconidia ranged between 22–51 μm having 2–7 septa. Microconidial size was highly variable between 6–30 μm. Nine isolates from cluster III exhibited purple to dark purple pigmentation, purple to light purple colony colour, and fluffy to less fluffy mycelium. The size of macroconidia was between 18–40 μm having 2–7 septa. The virulent isolates Raichur and Davanagere belonged to this cluster having pointed and blunt conidia respectively. Seven isolates from cluster IV exhibited purple pigmentation, except F32 and G1-3 exhibited yellow and pink pigmentation, respectively, white to dirty white and light purple colony colour, fluffy to less fluffy, and appressed mycelium. Pointed shape macroconidial size was in the range of 26 to 32 μm with 2–3 septa. The FUG-7 isolates had 6 septa. All isolates in this cluster were pointed in shape. The size of microconidia was in the range of 11–22 μm. Eleven isolates form cluster V having purple pigment, white to dirty-white colony with fluffy growth. The size of sickle-shaped macroconidia was from 22 to as large as 53 μm with up to 2–7 septa. Microconidial size was in the range of 10–21 μm. The virulent isolate F13 belonged to this cluster. Nine isolates from the VI cluster contained variable pigments of pink, yellow, white, and orange. Mycelial colour was white to dirty white, and fluffy mycelium. Macroconidial size was in the range of 21 to 44 μm with 2–6 septa. Microconidial size was in the range of 10–26 μm. The FUG8 had large size conidia with 4 septa. Cluster VII has seven isolates having variable pigment range. Isolates belonging this cluster had white to dirty white and light purple colony colour, and fluffy and dense mycelium. Macroconidial size was in the range of 21–36 μm with 2–7 septa. The virulent isolate FUG15 had the largest conidia in this cluster with seven septa. In cluster VIII, eleven isolates exhibited purple pigmentation with white and dirty white colony coloration and fluffy mycelium. This cluster had larger sickle-shaped conidia with 20 to 44 μm with as high as 8 septa. Microconidial size was in the range of 17 to 20 μm. Six isolates in cluster IX exhibited purple to dark purple pigmentation, having purple to light purple colony colour, fluffy mycelium. The size of macroconidia was in the range of 22–45 μm and 2–6 septa. This cluster consisted of pointed and blunt-end conidia. This cluster had a maximum of 3 virulent isolates including F18, FUR 11, and F59 (Figure 3).

**FIGURE 4:**
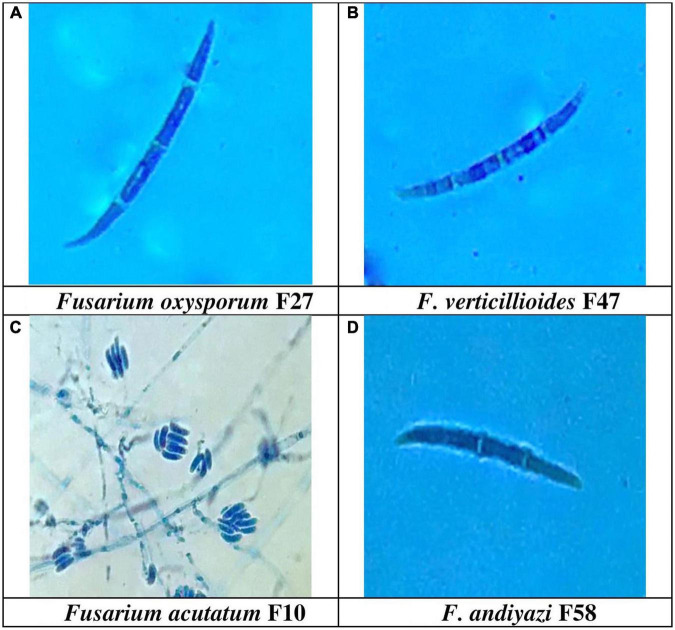
Conidial morphology of *Fusarium* spp. **(A)** Sickel-shaped Macroconidia of *Fusarium oxysporum* (50.3 μm ± 17.5 × 1.7 μm ± 0.8) 1000X, **(B)**. Sickle-shaped *F. verticillioides* (40.0 μm ± 1.0 × 2.3 μm ± 1.0) 1000X, **(C)** False head and monophialide conidiogenesis of *F. acutatum*, 400 X, and **(D)** Blunt ended macroconidia of *F. andiyazi* (18.0 μm ± 2.6 × 2.0 μm ± 1.3) 1000 X observed in the present study.

The production of microconidia occurred in chains and branches upon being grown on the SNA media. On a CILIKA™ digital microscope, the length and width of randomly selected microconidia and macroconidia were measured. Macroconidia produced three distinct types of spores, including those with sickle- or tapering-shaped, pointy, and blunted ends. There was a greater formation of sickle-shaped macroconidia in 32 *Fusarium* isolates, while 30 isolates produced pointed macroconidia, and five isolates produced blunt macroconidia. Sickle-shaped macroconidia ranged in size from 53.0 μm ± 8.0 × 1.5 μm ± 0.5 m (FUR 13) with a Quotient value (Qv) of 35 μm ± 7.4 to 13 μm ± 2.5 × 2 ± 1.0 m (F12) with a Qv of 7 ± 2.6. Comparatively, blunt-shaped macroconidia are smaller, ranging between 30.0 μm ± 3.0 × 2.5 μm ± 1.0 m (FUR 11) Qv 12.0 ± 4.4 to 18.0 ± 2.6 × 2.0 ± 1.3 μm (F 58). The size of pointed macroconidia ranged from 44 μm ± 3.5 × 1.5 μm ± 0.5 m (F52) Qv 29.0 ± 8.4 to 13.0 ± 2.0 × 2.0 ± 0.5 m (Qv 6.5 ± 1.3) (F48). Microconidial dimensions ranged from 23.0 μm ± 3.1 × 2.5 μm ± 0.5 m (F8) Qv 9.3 ± 0.7 to 5.0 ± 1.5 × 1.5 ± 0.5 m (F48) Qv 3.6 ± 0.3 (Supplementary Table 2). The number of septa detected in macroconidia varies between 2 and 8 septations. With 0-1 septa, microconidia were circular to oval in shape. The F52, F13, FUR 15, F16, F22, F14, F22 (D2), and B1-1are examples of isolates with 7 septa in their macroconidia, while only isolate F20 was reported to have 8 septa. The range of macroconidia is 10–53 μm × 1–4 μm. Microconidia size ranges from 4–31 μm × 1–3 μm. The *F. acutatum* isolate FUR 13 had macroconidia of size 53.0 μm ± 8.0 × 1.5 μm ± 0.5, Isolate F13 developed conidia with lengths of sizes 51.0 μm ± 15.4 × 2.0 μm ± 0.5. The *F. oxysporum* isolate F27 had large macroconidia 50.0 μm ± 17.5 × 1.7 μm ± 0.8 with Qv of 30.2 ± 14.9 (Figure 4).

### *In-vitro* pathogenicity evaluation of *Fusarium* isolates

#### Effect of treatment with *Fusarium* spp. on maize seed germination

**FIGURE 5:**
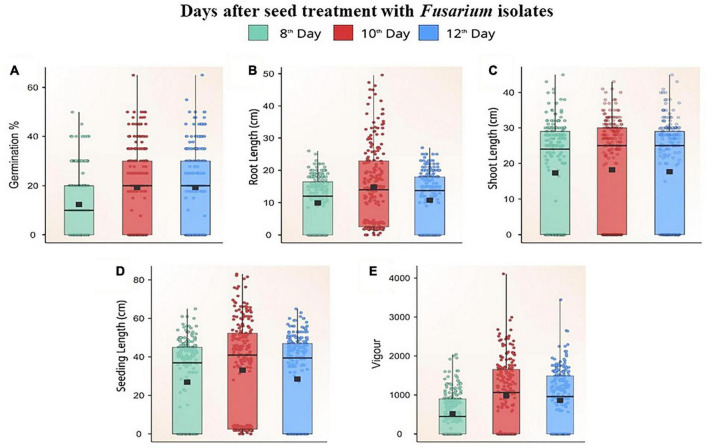
Box plot showing the distribution of germination percent **(A)**, root length **(B)**, shoot length **(C)**, seedling length **(D)**, and vigour **(E)** on different days after challenging maize seeds with *Fusarium* isolates.

*Fusarium* isolates significantly altered the seed germination of maize, the germination rate of different *Fusarium* spp. treated maize seeds varied between 0 and 70% on different days following fungal inoculation. Based on the extent of germination, these isolates were categorized as less virulent (F39, F7, FUG1, F49, F58, F32, F19, Davanagere), moderately virulent [FUR13, F28, F52, FUG16, F38, F45, Mandya, F3, F44, F13, Chokhla, F1, F57, FUG6, F47, F43, F22 (D2), F8, Mandya 2 and Haveri]. Virulent isolates F46, F12, F21, F9, F11, FUR50, F31, F35, FUG4, FUG3, F9, FUG8, F14, F10, F20, F42, FUG7, F48, F6, FUG5, F4, F55, F59, F26, Mysore, Raichur, and F36 inhibited germination of maize seeds on a paper towel by one hundred percent. Non-virulent isolates consistently promoted germination up to 12 days after inoculation (*p* ≤ 0.05), but virulent isolates inhibited germination. The maximum rise in germination was found 10 days after inoculation, as indicated by the Box Plot trend for change in germination percentage (*p* ≤ 0.05; Figure 5A). The non-germinating seeds were determined to be decaying or weakly germinated.

#### Effect of treatment with *Fusarium* spp. on maize seedling root length

Based on the pathogen’s virulence, there was a significant (*p* ≤ 0.05) difference in root length between *Fusarium* spp.-treated seeds and untreated control seeds. The treated seeds had a mean root length between 0–24.8 cm. Seeds inoculated with *Fusarium* spp. isolates showed a steady increase in root length for the first 10 days, and, thereafter, a decline (Figure 5B). The untreated seeds produced roots measuring 25 cm in length. The roots inoculated with isolates of reduced virulence [F52, F38, Mandya, F44, Chokhla, F1, FUG, FUR12, F16, F49, F43, F19, F22 (D2) Mandya 2, Davanagere] grew relatively vigorously. These isolates have the potential to reduce root length by up to 20% compared to the control. FUG1, F16, F44, FUG10, F43, F19, FUR12, F1, F38, Davanagere, FUG16, FUR13, F27, FUG9, F13, FUG2, F47, G1-3, F39, F34, FUR14, F8, F57, F18, F34, FUG6, F7, F3, F2, F45, F58, B1-1, F22, F28, and F25 were the isolates which reduced root length by 22 to 60 %. The remaining isolates belonged to a very virulent group that caused root lengths of fewer than 10 cm. W3-2, F36, Raichur, Mysore, F26, F59, F55, F4, FUG5, F6, F48, FUG 7, F42, F20, F10, F14, FUG 8, F9, FUG3, FUG4, F35, F31, FUR 15, F11, F21, F12, and F46 were the isolates that produced a substantial reduction in root length (Supplementary Table 3). Other than W3-2, all these isolates inhibited seed germination. Thus, these isolates reduced root length by 100%t and are considered highly virulent isolates. Data points in the Box plot depict the reduction in root length on the 12^th^ day after treatment with all *Fusarium* spp. (Figure 5B).

#### Effect of treatment with *Fusarium* spp. on maize seedling shoot length

The shoot length of the seedlings was measured at 8, 10, and 12 days after treatment with *Fusarium* spp. isolates. The variation in the shoot length between three different periods of the evaluation was not significant. There was a correlation between the seedlings’ length and the isolates’ virulence. The greater the length of the seedling, the less virulent the inoculated *Fusarium* isolate. The shoot length of the seedlings varied significantly, ranging from 0 to 43 cm. The shoot length of the seedling treated with the F38 *Fusarium* isolate F38 was 42.1 cm which was at par with the untreated control having a shoot length of 45 cm. The shoot length inoculated with the studied isolates F52, FUR14, F19, F38, Mandya, F3, F1, F18, F22 (D2), F22 (2C), B1-1, F47, and F43 ranged between 30 to 43 cm which caused a 6.4 to 32.1% reduction in shoot length as compared to untreated control (Figure 5C). Thus, these isolates were considered less virulent. The average shoot length of seedlings treated with F8, F34, F22 (2C), FUG1, FUG 16, FUG 9, FUG10, FUG2, F45, FUR11, F57, G1-3, Mandya 2, Chokhla, F27, F34, F44, F16, F39, F7, F45, F58, F49, Haveri, F32, F2, W3-2, FUR 13, F32, F33, and Davanagere, ranged between 20–29.9 cm. Thereby recording a 33.8 to 55.4% reduction in shoot length and referred to as moderately virulent isolates (Figure 5C). The drastically shorter shoot length ranged between 0−19.6 cm, caused by the *Fusarium* spp. isolates FUG2, F28, F25, F12, F21, F9, F11, FUR15, F31, F35, FUG4, FUG3, FUG8, F14, F10, F20, F42, FUG7, F48, F6, FUG5, F4, F55, F59, F26, Mysore, Raichur, and F36 thereby causing 56.3–100 % reduction in shoot length (Supplementary Table 4). Thus, these isolates were deemed the most virulent isolates. Except for three isolates (FUG2, F28, and F25), the remaining 25 isolates did not permit the germination of seeds.

#### Effect of treatment with *Fusarium* spp. on maize seedling vigour

According to the measurements of seed germination, shoot length, and root length, the seedling vigour of each isolate of *Fusarium* spp. there were considerable differences in germination and shoot-root length, resulting in significant differences in seedling vigour. Using the formula given in the materials and methods, the vigour of seedlings was estimated. The untreated control seeds produced the most vigorous seedlings with a vigour index of 2347 ± 10.86. Isolates of *Fusarium* spp. with a high vigour index were rated less virulent. F44, F7, F3, FUG16, F39, Davanagere, F13, F49, FUG 1, F58, F32, and F19 which had a vigour index of more than 1,500, were responsible for up to 36% decrease in vigour compared to the control (Figures 5D, E). The 29 moderately virulent isolates had a vigour index between 822.6 ± 9.94 and 1463.9 ± 9.53. These isolates are F34, F25, F27, B1-1, G1-3, F18, F22 (D2), FUR14, FUG9, F47, Mandya 2, F52, F16, F22 (2C), FUG10, F43, F7, F8, F57, F45, F28, FUR13, FUR12, Chokhla, FUG6, Mandya, F1, FUR11 and F38 (Supplementary Table 5). These isolates reduced seedling vigour by 37.6 to 64.9 % in an *in vitro* paper towel test. The 30 isolates were recorded to cause 65–100 % reduction in seedling vigour index with a vigour index from 822.2 ± 9.12 to 0. These 30 isolates F46, F12, F21, F11, FUR15, F31, F35, FUG4, FUG3, F9, FUG8, F14, F10, F20, F42, FUG7, F48, F6, FUG5, F4, F59, F26, Mysuru, Raichur, F36, F55, W3-2, FUG2, F33, and F2 with least vigour index were considered as most virulent (Supplementary Table 5). Data points in the box plot depict that higher vigour was observed 10 days after seed treatment with *Fusarium* isolates. The median of boxes on 10^th^ and 12^th^ day after inoculation was near 1,000. Our observations showed that 38 isolates were below the median line with the vigour index of less than 1,000 while 33 isolates were above the median line with a vigour index more than 1000 (Figure 5E).

#### Pathogenicity of *Fusarium* isolates during Kharif 2020 and *Rabi* 2020-21 seasons under field conditions

**FIGURE 6:**
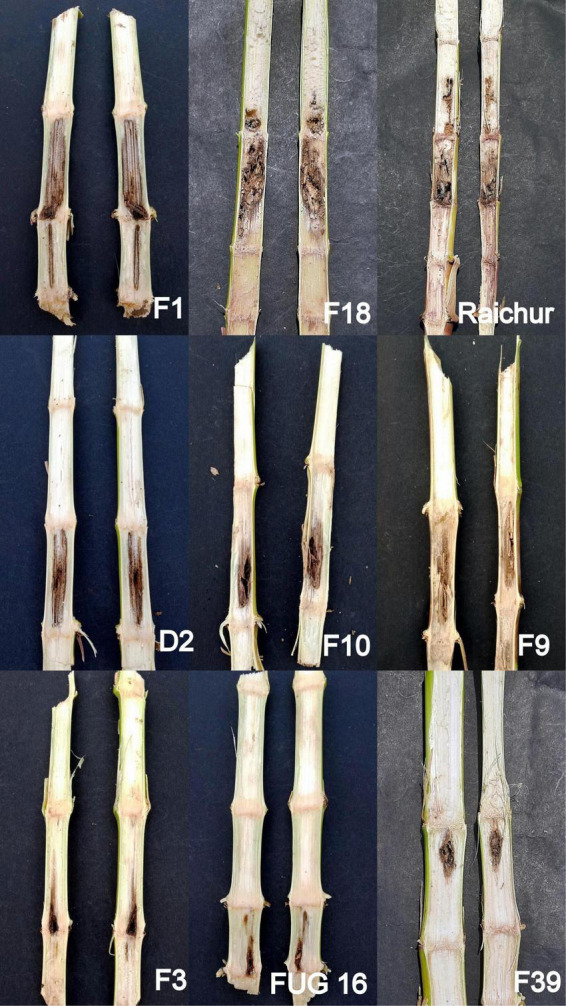
Comparative lesion size of virulent (F1,F18, Raichur), Moderately virulent (D2, F10, FUG49), and less virulent (F3, FUG 16, F39). Virulent isolates caused lesions covering 2–3 nodes, moderately virulent isolates covered entire pith between nodes, and less virulent isolates a lesion restricted to the inoculated area.

**FIGURE 7:**
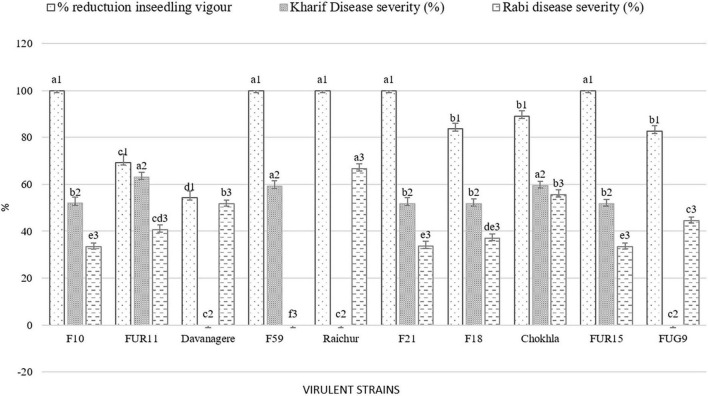
Comparative virulence among *Fusarium* isolates observed during the *in-vitro*, Kharif 2020 and Rabi 2020-21 season. Values for histograms sharing the same letter label are not significantly different (*P* > 0.05).

The pathogenicity of sixty *Fusarium* isolates was evaluated with the development of visible symptoms such as drooping, wilting, and drying of leaves, empty cob development, and an increase in the angle between stalks and cobs in the field. All the isolates were able to cause infection. The intensity of disease symptoms varied among the isolates. At the harvest, all the inoculated plants were split open to measure lesion length (Figure 6). During the Kharif 2020 season, out of 60 isolates, nine were less virulent (11–25% PDI, average lesion length = 2.83–6 cm), 39 were moderately virulent (25–50% PDI, average lesion length = 5.5–12.03), and 12 were virulent (50–67% PDI, average lesion length = 6–14 cm; Figure 7A). The virulent isolates F21, FUR15, F18, F35, F1, F32, Chokhla, FUR12, F59, F25, FUR11, F13; exhibited virulent reactions with mean severity ranging from 4.7 to 6 cm lesion length (50 to 67% PDI). Isolates FUR14, FUG16, FUR13, F38, F27, F3, F43, FUG7, and F2 were less virulent or avirulent, with mean severity ranging from 1–2 (11–25% PDI). The F25, F35, and F59 were found to be virulent both *in vitro* and in the field during the *Kharif* season (Supplementary Table 5). There were 20% virulent isolates, 65% moderately virulent isolates, and 15% less virulent isolates. During *Rabi* season 2020-21, nearly 60 isolates, including nine distinct *Fusarium* isolates, were obtained from South India during this season and were evaluated for pathogenicity (Davanagere, Mysore, Mandya, Mandya 2, Raichur, Haveri, B1-1, W3-2, and G1-3). The *Kharif* season isolates with a lower PDI and severity of symptoms were eliminated during the *Rabi* season (FUR14, FUG16, FUR13, F38, F27, F3, F2, F43, and FUG7). Isolates with mean disease severity in the range of 19–22% and causing lesion length of 2–2.6 cm (FUG2, FUG1, F44, F52, FUR10, F39, FUR12, Mandya 2) were deemed to be less virulent. As many as 47 isolates were moderately virulent with the mean disease severity of these isolates ranging between 25.9 and 48.1 % and lesion lengths ranging between 2.3 and 4.3 cm (Supplementary Table 5). The Raichur, Chokhla, Davanagere, F1 and F13 isolates have an average symptom severity ranging between 51.9 and 66.7% and were considered virulent isolates (Figure 7B; Supplementary Table 5). Three isolates, F1, Chokhla, and F13, were virulent throughout the *Kharif* and *Rabi* seasons. In the *Rabi* season, virulent isolates like F35, F21, F18, FUR15, F25, F32, and FUR11 from the previous season were determined to be moderately virulent. During the *Rabi* season, five isolates F13, F1, Davanagere, Chokhla, and Raichur were found to be virulent. Of the total analysed isolates 8% were virulent, 77% were moderately virulent, and 15% were less virulent during *Rabi* season (Figure 7B). The inoculated stem showed vascular discoloration, from where the pathogen was reisolated to confirm Koch’s postulates and to confirm the similarity of cultural and microscopic morphology with those of the inoculated *Fusarium* isolates.

#### Comparative severity *in vitro*, in *Kharif* and *Rabi* seasons

**FIGURE 8:**
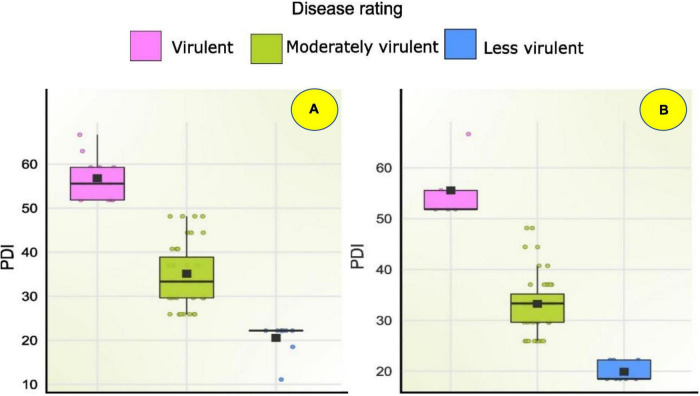
Box plot showing disease severity levels of *Fusarium* spp. isolates during **(A)** Kharif 2020 and **(B)** Rabi 2020-21; Within each box horizontal black line denote median values.

Seventy-one *Fusarium* isolates were tested *in vitro* using the paper towel method. In both the *Kharif* and *Rabi* seasons, the pathogenicity of sixty *Fusarium* isolates was evaluated using the toothpick inoculation method. The *in vitro* investigation revealed that, of the 71 isolates examined for pathogenicity, 30 isolates inhibited germination and decreased seedling growth. Based on less vigour of the seedlings, these isolates were considered virulent isolates. Under *in vitro* conditions, as mentioned above F46, F12, F21, F11, FUR15, F31, F35, FUG4, FUG3, F9, FUG8, F14, F10, F20, F42, FUG7, F48, F6, FUG5, F4, F59, F26, Mysore, Raichur, and F36, exhibited a 100% drop in seedling vigour, whereas F55, W3-2, FUG2, F33, and F2 exhibited a reduction in vigour in the range of 65 to 99.5% (Supplementary Table 5). While the *in vitro* pathogenicity of the isolates was determined by their ability to diminish seedling vigour, the virulence of the field-level evaluation was determined by the severity of the disease. Pathogenicity evaluation of the 60 isolates during Kharif 2020 reported 12 virulent isolates with disease severity of more than 50% including F21 (52 ± 0.58), F18 (52 ± 0.58), FUR15 (52 ± 0.58), F13 (52 ± 0.58), F1 (55 ± 0.58), F25 (55 ± 0.58), FUR12 (55 ± 1.15), F59 (59 ± 1.15), Chokhla (59.3 ± 1.15), F32 (59.3 ± 0.58), FUR 11 (63.0 ± 0.58), and F35 (66.7 ± 1.15). During the *Rabi* 2020-21 season experiment of pathogenicity evaluation, only five isolates exhibited disease severity above 50%. Those include F13 (52 ± 0.58), F1 (52 ± 1.15), Davanagere (52 ± 0.58), Chokhla (55.6 ± 1.15), and Raichur (67 ± 0.02) (Supplementary Table 5). Based on their performance in *in-vitro*, *Kharif*, and *Rabi* field studies, the ten most virulent isolates were selected for resistance evaluation of inbred lines. These isolates were F10, FUR11, Davanagere, F59, Raichur, F21, F18, Chokhla, FUR15 and FUG9 (Figure 8).

#### Molecular characterization of *Fusarium* spp. isolates

**FIGURE 9:**
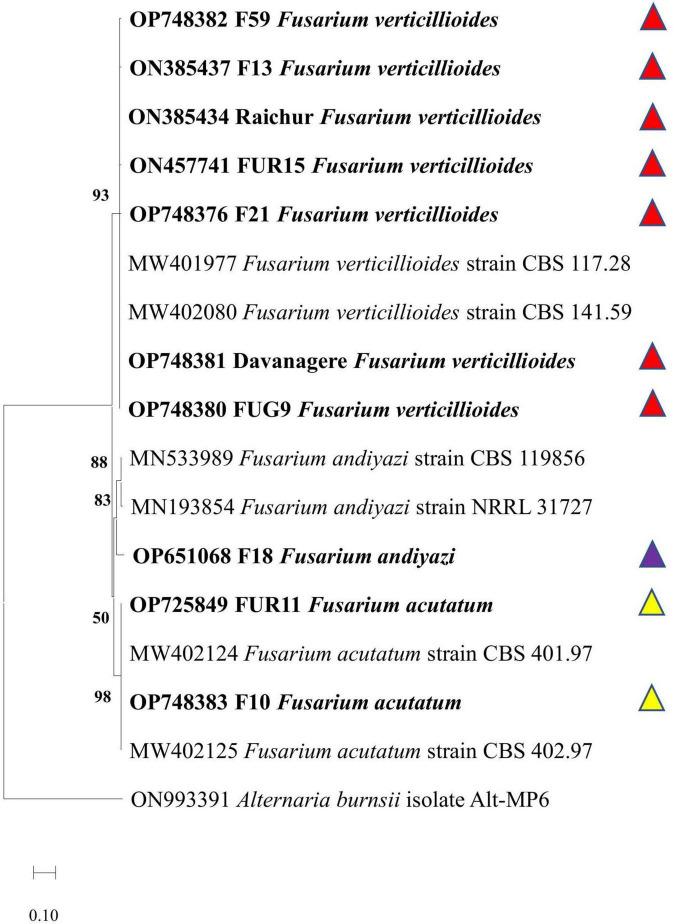
Phylogenetic analysis of *Fusarium* spp. virulent strains based on translation elongation factor (Tef-1α) sequences by MEGA X software using neighbour joining method with 1,000 bootstrap replication. Accessions in bold are strains characterized in the present experiment. Reference sequences were obtained from Crous et al. (2021). Two reference strains for each species are: *Fusarium verticillioides* (Reference species: MW401977 CBS 117.28 strain, MW402080 CBS 141.59 strain), *F. andiyazi* (Reference species: MN533989 CBS 119856 strain, MN193854 NRRL 31727 strain), and *F. acutatum* (Reference species: MW402124 CBS 401.97 strain, MW402125 CBS 402.97 strain). The *Alternaria burnsii* isolate Alt-MP6 (Sequence ID: ON993391) was used as an outgroup. 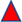 *Fusarium verticillioides*; 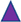 *Fusarium andiyazi*; 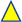 *Fusarium acutatum*.

The Tef 1α partial gene sequences of the ten most virulent isolates were searched for closest homologies in the NCBI. Based on the reference sequences of the closest species a phylogenetic tree was constructed. The ten most virulent isolates were identified as strains of *Fusarium acutatum* (FUR11, F10), *Fusarium verticillioides* (Syn. *Gibberella fujikuroi* var. *moniliformis*) (Davanagere, Raichur, FUG9, F13, FUR15, F21, and F59), and *Fusarium andiyazi* (F18). The deposited GenBank accession numbers of the respective isolates (or strains) were OP725849, OP748381, ON385434, OP748380, ON385437, ON457741, OP748376, OP748376, OP748382, OP748383, and OP651068. The phylogenetic tree demonstrated that the virulent strains recovered from different locations in India with similar species identity clustered together, and confirmed the homology-based identification by forming three distinct clades with *Fusarium verticillioides Fusarium verticillioides* (Reference species: MW402103 CBS 181.31 strain, KF499582 CBS 218.76 strain), *F. andiyazi* (Reference species: MN533989 CBS119856 strain, OP486865 LLC1194 strain), and *F. acutatum* (Reference species: KR071754 CBS 402.97 strain, MW401971 CBS 113964 strain) (Figure 9).

## Discussion

*Fusarium* spp. are major pathogen of maize causing symptoms at different growth stages such as crown root rot, seedling rot, stalk rot, wilt, and ear rot of maize in many countries. Among various growth-stage diseases, PFSR which include *Fusarium* stalk rot, charcoal rot, and late wilt is a major threat to the cultivation of maize crop that affects crop just after tasselling and cob-filling stage in different parts of the world including India, leading to severe yield losses and reduction in grain quality (Payak and Sharma, 1983; Krishna et al., 2013). Frequent incidences of this disease have been reported in many Indian states such as Rajasthan, Gujarat, Madhya Pradesh, Karnataka, Telangana, and Andhra Pradesh. Moreover, recent incidences of PFSR are also observed in Punjab and Maharashtra. Due to the increasing incidences of this fungal pathogen and the extent of pathogenicity in maize crops, we designed a study to thoroughly investigate the morphological diversity and screen the pathogenic potential of *Fusarium* spp. causing PFSR in various Indian states having different agro-climatic conditions.

Changes in agro-climatic conditions alter the attributes of the fungus and can drive the emergence of novel, and climate-resilient fungal species with negative consequences for food security (Shekhar and Singh, 2021). Our study began with a survey of different PFSR-infected maize fields to cover diverse locations with different agro-climatic conditions of Indian states where 71 *Fusarium* spp. were isolated from symptomatic maize stalks from five agro-climatic zones *viz*. Eastern plateau and hills, Central Plateau and hills, Western plateau and hills, Southern plateau and hills, and Gujarat plain and hills. Variations in colony appearance were observed between the isolates, such as mycelial variations such as fluffy and sparse white growth, and white and purple mycelial growth having a range of colour vacations such as violet, light violet, light pink, and light pink to light violet, dark violet, and filthy white. In a previous study, authors also observed pink, violet, purple, and brown mycelial colours at later stages on PDA plates (Pavlovic et al., 2016). Harleen et al. (2016) investigated the morphological characteristics and distribution of 56 *Fusarium* isolates from various maize-growing locations in Punjab. Ramesha and Naik (2017) examined the colony morphology and pathogenicity of *Fusarium* isolates from Karnataka. Mamatha et al. (2020) surveyed 13 Telangana villages and evaluated the incidence of post-flowering stalk rot and the morphological variety of *Fusarium* spp. Traditional studies of cultural characteristics and microscopic features of micro and macroconidia and chlamydospores as well as the presence or absence of other morphological structures assist in taxonomic classification and identification of *Fusarium* species. The morphological characteristics of complex *Fusarium* spp. are challenging to observe. Thus, from the phenotype dendrogram (Figure 3) the *F. verticillioides* strains were observed to spread throughout the various clades. Moreover, the *F. acutatum* and *F. verticillioides* strains appeared in the same clades.

Considering these variations in morphological features among strains it is very difficult to identify strains. Molecular techniques help in a more reliable identification of species, but it is pertinent to complement classical methods. Sporulation of micro and macroconidia was observed on SNA media in the range of sickle- or tapering-shaped, pointy, and blunted ends. Nevertheless, several researchers obtained comparable results (Varela et al., 2013; Shin et al., 2014; Zhang et al., 2021). Ramesha and Naik (2017) found that macro-conidia was sickle-shaped, pedicellate, and distributed, with a high degree of morphological variation in terms of the shape of the micro and macroconidia, as well as variation in the number of septa in each strain and micro conidiophore branching.

*Fusarium verticillioides* which was predominantly isolated from the stalk rot tissues formed long slender macroconidia having a curved apical cell to a tapered point with basal cell notched hill bearing 2–5 septa. Sempere and Santamarina (2009) also observed similar conidial features with false head formation. Based on multivariate cluster analysis of morphological characteristics, 71 isolates were grouped in 9 clusters. Some of the virulent isolates were molecularly characterized and confirmed the identity. In our investigations, out of the ten most virulent isolates, 7 were identified as *F. verticillioides*. This showed that *F. verticillioides* is the major pathogen causing PFSR. Our results support the previous finding that *F. verticillioides* is the dominant *Fusarium* spp causing PFSR in India (Swamy et al., 2019; Jambhulkar et al., 2022). Among the virulent strain *F. verticillioides* (Davanagere, Raichur, F13, FUG9, FUR15, F21, and F59), except the FUG9 and FUR 15, remaining strains had purple to dark purple pigmentation. FUG9 and FUR15 were in close proximity but differ in sub-clade due to change in pigmentation and shape of conidia. Davanagere, Raichur and F59 had light pink to light purple colony colour, remaining strains had white to dull white colony colour. Largest among seven *F. verticillioides* strain was F13 and smallest was F21. Maximum number of septa were observed in F13 and FUR15 while minimum number observed was in F21. Two strains of *F. acutatum* harboured variation in morphology and micrometry still molecular characterization identified them as one strain of *F. acutatum*. These variations in cultural morphology and micrometry did not lead to accurate identification of species. Such variation in the cultural morphology of *F. verticillioides* was reported by previous researchers (Prasad and Padwick, 1939; Patil et al., 2007; Harleen et al., 2016).

The pathogenicity of 71 isolates was evaluated in an *in-vitro* test and subsequently, 60 isolates in *Kharif* and *Rabi* field experiments. The present study was the first nationwide evaluation of the pathogenic variability of *Fusarium* spp. isolated from six states and five agroclimatic zones of India. Pathogenic variabilities among isolates of *Fusarium* spp. were ascertained based on each isolate causing lesions at the inoculated node and temporal variations in appearance lesions. *Fusarium* isolates exhibit brown to black discoloration, whereas sterile toothpicks are less infected (Yu et al., 2017). Mesterhazy et al. (2020) evaluated resistance to *F. graminearum*, *F. culmorum*, and *F. verticillioides* through toothpick inoculation and toxin response. The toothpick inoculation technique yielded accurate and efficient pathogenicity results, and discovered more genotypic diversity. The results of the pathogenicity tests revealed that *Fusarium* spp. isolates were less virulent to highly virulent against cv. Pusa Composite 4. Kharif 2020 had the highest infection rate of the two experimental years, possibly due to meteorological conditions. Similar to our findings, pathogenic potentials of 56 *F. verticillioides* isolates during *Kharif* and spring seasons on maize were documented in Punjab (Harleen et al., 2016). Ramesha and Naik (2017) proved the pathogenicity of *Fusarium* isolates to produce disease symptoms with the development of blackening of vascular bundles in Karnataka. There are claims that *Fusarium* spp. inoculums enhance root growth without compromising germination (Roman et al., 2020), but we noticed a reduction in root growth using the paper towel approach. There are reports of a constant decline in maize root length following *Fusarium* spp. inoculation (Ye et al., 2013; Kuhnem et al., 2015). Hassani et al. (2019) discovered that natural infection with *Fusarium* spp. decreases shoot and root length. Similar outcomes were reported under *in-vitro* circumstances in the present study. *In vitro* pathogenicity evaluation reported 30 virulent isolates with seedling vigour index in the range of 0 to 822.2 which suggested that there was a 65 to 100% reduction in seedling vigour index. Only 8 percent of the isolates with a vigour index of more than 1,500 remained avirulent. Significant differences in stalk rot index were observed among 71 isolates. *In vitro* inoculation with *F. moniliforme* and *Aspergillus niger* showed not only a pathogenic effect on the germination of maize seeds but also retarded seedling growth (Hussain et al., 2013).

The pathogenic effect of *Fusarium* spp. isolates from different agroclimatic zones showed variable pathogen virulence in field experiments which ultimately led to symptoms such as drooping, wilting, drying of leaves, empty cob development, and increased angle between stalks and cobs in the field. The virulence of *Fusarium* species isolates Raichur (66.6%), and F35 (66.7%) were the highest. Under *in vitro* conditions, FUR15, F35, F59, Raichur, and F21 were considered highly virulent isolates. In the *Kharif* season, FUR11 (62.9%), Chokhla (59.3%), F59 (59.2%), F1 (55.5%), F18 (51.8 %), F21 (51.8%), F13 (51.8%), and in *Rabi* season Raichur (66.6%) and Chokhla (55.6%) were reported as most virulent. In this regard, some reports suggest that virulency may differ in different agroclimatic zones (Olowe et al., 2018). The most virulent isolates were from the eastern side of the Central Plateau and hills, the northern part of the Western plateau, and hills. The eastern side of the Central Plateau and hills is a hot, humid zone of southern Rajasthan. Therefore, the greatest observed incidence of the pathogen virulence was predominant in southern Rajasthan, suggesting that PFSR is the most important disease in maize. High temperature and relative humidity of southern Rajasthan than in other Rabi maize-growing states make it a hot spot for PFSR in maize. Thus, such climatic conditions are responsible for causing significant economic loss by PFSR in the eastern side of the Central Plateau and hills, the northern part of the Western plateau and hills, and southern plains and hills as compared to other agroclimatic zones (Czembor et al., 2015; Jambhulkar et al., 2022).

Taken together, in the present study, we compared the disease index upon inoculations with 60 isolates of *Fusarium* spp. collected from geographically diverse states of India, *in vitro* and in the *Kharif* and *Rabi* seasons. Ten isolates recorded the highest disease index during pathogenicity evaluations and were considered the most virulent isolates. Those isolates were molecularly identified as strains of *Fusarium acutatum* (FUR11, F10), *Fusarium verticillioides* (Davanagere, Raichur, FUG9, F13, FUR15, F21, and F59), and *Fusarium andiyazi* (F18). Due to the pronounced intraspecies morphological variabilities and the presence of multiple species within the *F. fujikuroi* Species Complex (Yilmaz et al., 2021), sequencing of Tef 1-α is recommended to reliably identify *Fusarium* pathogens, and future studies should include this information as a baseline before making recommendations in guiding pathologists for disease management. Moreover, the respective *Fusarium* isolates will be used in our program to evaluate the resistance of different maize inbred lines as a critical component of PFSR disease management.

## Data availability statement

The data presented in the study are deposited in the NCBI GenBank repository, accession numbers: OP725849, OP748381, ON385434, OP748380, ON385437, ON457741, OP748376, OP748382, OP748383, and OP651068.

## Author contributions

PJ conceived and obtained funding from DST-SERB. PJ and JH collected isolates from diseased plants from different agroclimatic regions of India and prepared the first draft of the manuscript. JH performed morphological characterization, microscopy and did the phylogenetic analysis. PJ, JH, and RB did pathogenicity. RB and JH did molecular characterization and carried out clustering. PB, PJ, and DL edited the manuscript. MA, SC, DL, and AK provided valuable insights into the manuscript. All authors approved the final version of the manuscript.

## Footnotes

1
https://www.ncbi.nlm.nih.gov/
