# Supplementary material for: Morphological characterization, pathogenicity screening, and molecular identification of Fusarium spp. isolates causing post-flowering stalk rot in maize
Source: Front Microbiol. 2023 Mar 31;14:1121781. doi: 10.3389/fmicb.2023.1121781 (PMC10102488; doi:10.3389/fmicb.2023.1121781)
Supplement: Supplementary file 1 [file Table_1.DOCX]

**Supplementary files**

**Supplementary Table 1.** Geographical locations of *Fusarium* spp. isolates collection

| **Sr. No.** | **Isolate** | **Location** | **Latitude** | **Longitude** |
| --- | --- | --- | --- | --- |
|  | F1 | Peepalkhunth | 23° 46' 9.3324'' N | 74° 38' 50.9244'' E |
|  | F20 | Ranapur MP | 22° 36' 21.2292'' N | 74° 32' 56.706'' E |
|  | F39 | Palodara, Banswara | 23° 30' 32.0544'' N | 74° 24' 16.1316'' E |
|  | F25 | Bodla Banswara | 23° 29' 31.416'' N | 74° 23' 21.7104'' E |
|  | F19 | Siapur Banswara | 23° 28' 41.2536'' N | 74° 23' 24.8028'' E |
|  | F43 | Leemthan Bsw | 23° 27' 20.7576'' N | 74° 24' 58.1868'' E |
|  | F12 | Nawagaon Bsw | 23° 29' 32.2656'' N | 74° 26' 16.1088'' E |
|  | F10 | Lakshmipura Bsw | 23° 27' 10.8396'' N | 74° 20' 54.5244'' E |
|  | F16 | Gangartalai | 23° 15' 33.5736'' N | 74° 10' 39.9756'' E |
|  | F34 | Anandpuri | 23° 22' 51.6828'' N | 74° 1' 53.688'' E |
|  | F27 | Naharpura | 23° 26' 18.1104'' N | 74° 7' 10.8732'' E |
|  | F28 | Masotia | 23° 36' 0.6192'' N | 74° 19' 38.5572'' E |
|  | F14 | Devlia | 23° 34' 33.9672'' N | 74° 20' 35.4552'' E |
|  | F32 | Senavasa | 23° 39' 53.874'' N | 74° 23' 23.0496'' E |
|  | F18 | Ghatol | 23° 44' 45.2832'' N | 74° 25' 53.328'' E |
|  | F26 | Peepal khunt | 23° 48' 46.7568'' N | 74° 37' 49.4724'' E |
|  | F11 | Arnod Pratap garh | 23° 53' 29.3568'' N | 74° 49' 22.116'' E |
|  | F35 | Chotisadri | 24° 21' 39.0924'' N | 74° 44' 17.8476'' E |
|  | F13 | Dhariyawad | 24° 5' 4.9524'' N | 74° 31' 35.9364'' E |
|  | F3 | Galiyakot | 23° 28' 33.0348'' N | 73° 59' 15.6264'' E |
|  | F31 | Alirajpur MP | 22° 19' 28.8156'' N | 74° 23' 10.392'' E |
|  | F4 | Jhabua MP | 22° 43' 41.0052'' N | 74° 33' 38.1096'' E |
|  | F42 | Ranapur MP | 22° 39' 16.4592'' N | 74° 31' 58.6704'' E |
|  | F33 | Dahod GJ | 22° 47' 42.6228'' N | 74° 19' 21.7092'' E |
|  | F49 | Godhra GJ | 22° 47' 56.2956'' N | 73° 52' 13.9908'' E |
|  | F36 | Kalol GJ | 22° 39' 57.5172'' N | 73° 33' 16.074'' E |
|  | F38 | Jhalod GJ | 23° 5' 26.16'' N, | 74° 9' 13.32'' E |
|  | FUG1 | Kalol GJ | 22° 29' 22.2472'' N | 73° 36' 27.1425 E |
|  | FuG2 | Jhalod, GJ | 23° 5' 49.68'' N | 74° 9' 49.97'' E |
|  | FuG3 | Chota Udaipur, GJ | 22°23’3.42” N | 73°42’40.98” E |
|  | FuG4 | Halol, GJ | 22°29’39.38” N | 73°27’54.29” E |
|  | FuG5 | Dahod, GJ | 22°50’19.6” N | 74°4’51.20” E |
|  | FuG6 | Pavagadh, GJ | 22°26’17.63” N | 73°35’19.64” E |
|  | FuG7 | Chapaneri, GJ | 22°27’43.94” N | 73°31’0.59 E |
|  | FuG8 | Pavagarh, GJ | 22°25’43.94” N | 73°43’22.25” E |
|  | FuG9 | Kalol, GJ | 23°13’41.45” N | 72°35’17.84 E |
|  | FuR10 | Sagrod, RJ | 23°27’43.6” N | 74°22’17.19” E |
|  | FuR11 | Kushalgarh, RJ | 23°17’33.6” N | 74° 25’17.19” E |
|  | FuR12 | Kohala, RJ | 23°37’12.23” N | 74°22’14.97” E |
|  | FuR13 | Paloda, RJ | 23°44’12.23” N | 74°12’14.97” E |
|  | FuR14 | Halol, GJ, | 22°32’19.38 “N | 73°28’14.23” E |
|  | FuR15 | Banswara, RJ | 23°33’41.85” N | 74°28’14.97” E |
|  | FuG16 | Pavagarh, GJ | 22°28’ 17.76” N | 73°34’ 2.14” E |
|  | F44 | Santrampur GJ | 23° 11' 35.6244'' N | 73° 53' 14.1576'' E |
|  | F45 | Jhalod GJ | 23° 5' 21.714'' N | 74° 9' 23.3316'' E |
|  | F46 | Limdi GJ | 22° 58' 57.27'' N | 74° 9' 32.6304'' E |
|  | F47 | Godhra GJ | 22° 46' 36.9768'' N | 73° 30' 37.0872'' E |
|  | F48 | Wagdodia GJ | 22° 18' 37.9368'' N | 73° 25' 54.1992'' E |
|  | F2 | Garhi | 23° 33' 54.432'' N | 74° 8' 51.8496'' E |
|  | F21 | Banswara RJ | 23° 30' 38.8944" | 74° 22' 45.807" |
|  | Chokhla, | Chokhla, Banswara RJ | 23° 25' 6.2112'' N | 74° 19' 13.8864'' E |
|  | F52 | Nagawara Banswara | 23° 23' 5.5212'' N | 74° 18' 36.0036'' E |
|  | F55 | Gangartalai Banswara | 23° 15' 6.0948'' N | 74° 10' 30.1908'' E |
|  | F57 | Kushalgarh | 23° 10' 32.1168'' N | 74° 26' 27.2616'' E |
|  | F58 | Jhalod Gujrat | 23° 5' 34.7892'' N | 74° 11' 21.8508'' E |
|  | F59 | Dahod Gujrat | 22° 53' 26.4948'' N | 74° 19' 40.2096'' E |
|  | F6 | Karji Banswara | 23° 23' 39.7824'' N | 74° 22' 59.8116'' E |
|  | F7 | Sevniya | 23° 45' 50.7816'' N | 74° 26' 49.5096'' E |
|  | F8 | Ganoda Banswara | 23° 45' 42.4116'' N | 74° 16' 40.1412'' E |
|  | F9 | Galiyakot | 23° 32' 19.4856'' N | 74° 0' 49.5648'' E |
|  | F22(D2) | Halol GJ | 22° 30' 31.5036'' N | 73° 22' 42.8016'' E |
|  | F22 (2C) | Kalol GJ | 22° 30' 14.148'' N | 73° 25' 55.1568'' E |
|  | Davanagere | Davanagere | 14 ° 31 '49.6452' 'N | 75 ° 48 '3.9384' 'E |
|  | Mysuru | Mysuru | 12° 47' 10.4352'' N | 75° 12' 1.9404'' E |
|  | Mandya | Mandya | 12 ° 29 '34.3032' 'N | 76 ° 39 '51.4224' 'E |
|  | Raichur | Raichur | 16° 09' 31.54" N | 76° 31' 18.26" E |
|  | Haveri | Haveri | 14° 28' 12.00" N | 75° 22' 48.00" E |
|  | Mandya 2 | Mandya | 12° 23' 10.07" N | 77° 03' 34.34" E |
|  | Bagalkot B1-1 | Bagalkot | 16° 30' 15.5838" | 75° 17' 30.2706" |
|  | Warangal W3-2 | Warangal | 18° 0' 0.1980'' N | 79° 35' 17.3940'' E |
|  | Gokak G1-3 | Gokak | 16 ° 10 '0.1200' 'N | 74 ° 49 '59.8728' 'E |

**Supplementary Table 2:** Morphological features of *Fusarium* isolates

| **Isolate** | **Pigmentation** | **Pattern of mycelium** | **Type of mycelium** | **Colony colour** | **Macroconidia (µm)** | **Qv (Macro-condia)** | **Shape** | **Septation** | **Microconidia**  **(µm)** | **QV (Micro-conidia)** |
| --- | --- | --- | --- | --- | --- | --- | --- | --- | --- | --- |
| **F46** | purple | F to LF | SP | W to DW | 0 |  | 0 | 0 | 9.3±5.0×1.5±0.5 | 6.2±1.6 |
| **FUR13** | purple | F | C | W to DW | 53.0±8.0 ×1.5±0.5 | 35.33±7.4 | S | 2 to 5 | 21.0±1.0 ×2.5±0.5 | 8.4±1.3 |
| **F39** | purple | F | C | W to DW | 21.7±5.1 ×2.5±0.5 | 8.67±0.6 | S | 2 to 3 | 13.0±1.0 ×1.7±0.8 | 7.8±3.8 |
| **FUG9** | pinkish | F | C | W to DW | 28.3±4.5 ×2.5±0.5 | 11.33±0.5 | S | 2 to 6 | 14.0±2.0 ×1.7±0.8 | 8.4±5.4 |
| **F28** | orange | F | C | W to DW | 21.5±1.5 ×2±0.5 | 7.67±1.8 | P | 2 to 4 | **6.0±**1.0×1.0±0.5 | 6.0±3.0 |
| **F52** | purple | F | C | W to DW | 43.7±3.5 ×1.5±0.5 | 29.11±8.4 | P | 2 to **7** | 13.7±2.1 ×1.0±0.5 | 13.7±12.6 |
| **F12** | purple | F | C | LPU | 12.7±2.5 ×1.8±1.0 | 6.91±2.6 | S | 2 to 4 | 8.0±1.0 ×1.5±0.5 | 5.3±1.5 |
| **F21** | purple | F | C | W to DW | 22.7±4.2 ×2.5±0.5 | 9.07±0.5 | P | 2 | 12.3±2.5 ×1.5±0.5 | 8.2±2.9 |
| **FUR10** | purple | F | C | W to DW | 51.3±9.6 ×1.5±0.5 | 34.22±5.5 | S | 2 to 5 | 10.7±3.1 ×1.5±0.5 | 7.1±3.3 |
| **F7** | white | A | SP | LPU | 23.7±2.5 ×2.5±0.5 | 9.47±2.0 | S | 2 to 4 | **6.0**±1.0 ×1.2±0.3 | 5.1±1.5 |
| **FUR14** | pinkish | F | C | W to DW | 21.7±2.5 ×2.5±1.0 | 8.67±3.0 | S | 2 to 3 | 10.7±1.5 ×1.0±0.5 | 10.7±5.1 |
| **F22(2C)** | purple | F | C | PU | 16.7±3.1 ×2.5±0.5 | 6.67±1.9 | P | 2 | 10.0±1.0 ×1.5±0.5 | 6.7±1.8 |
| **FUG16** | orange | F | C | LP | 25.7±5.0 ×2.5±0.5 | 10.27±0.3 | S | 2 to 5 | 10.0±4.0 ×1.5±0.5 | 6.7±0.5 |
| **F38** | dark purple | F | C | PU | 13.7±2.5 ×2.0±0.5 | 6.83±1.2 | P | 2 | 8.0±4.6 ×1.5±0.5 | 5.3±1.3 |
| **F45** | purple | F | C | W to DW | 22.0±2.0 ×1.5±0.5 | 14.67±4.1 | S | 2 to 4 | 9.7±1.5 ×1.3±0.3 | 7.3±0.7 |
| **F25** | pinkish | F | C | W to DW | 23.3±1.5 ×1.5±0.5 | 15.56±4.9 | S | 2 | 10.7±3.1 ×1.5±0.5 | 7.1±2.2 |
| **Mandya** | purple | F to LF | C | W to DW | 18.0±3.6 ×2.5±0.5 | 7.20±0.4 | P | 2 to 3 | 11.3±1.5 ×1.5±0.6 | 7.4±4.1 |
| **F3** | purple | F | C | LPU | 16.3±1.5 ×2.0±0.5 | 8.17±1.4 | S | 2 | 9.3±2.5 ×1.5±0.5 | 6.2±1.8 |
| **F44** | purple | F | C | W to DW | 0 | 0 | 0 | 0 | 11.7±2.1 ×1.5±0.5 | 7.8±3.1 |
| **FUR11** | purple | F | C | Pu | 30.0±3.0 ×2.5±1.0 | 12.00±4.4 | B | 2 | 13.3±4.2 ×2.5±0.5 | 5.3±0.6 |
| **F13** | purple | F | C | W to DW | 51.0±15.4×2.0±0.5 | 25.50±2.4 | S | 2 to **7** | 13.7±1.5 ×2.0±0.5 | 6.8±1.9 |
| **Chokhla** | purple | F | C | W to DW | 0 | 0 | B |  | 14.7±4.5 ×1.5±0.5 | 9.8±5.2 |
| **F1** | dark purple | F | C | W to DW | 22.0±1.0 ×2.0±0.5 | 11.00±2.4 | B | 2 to 4 | 10.0±7.0 ×2.3±0.8 | 4.3±2.6 |
| **F11** | purple | F | C | W to DW | 19.7±1.5 ×2.0±0.5 | 9.83±2.0 | P | 2 | 14.7±3.1 ×1.2±0.3 | 12.6±4.4 |
| **FUR15** | orange | F | C | W to DW | 35.7±11.7 ×1.7±0.8 | 21.40±12.3 | P | 2 to **7** | 11.3±5.9 ×1.0±0.5 | 11.3±2.5 |
| **FUG1** | purple | F | C | W to DW | 21.7±1.5 ×2.0±0.5 | 10.83±2.3 | S | 2 to 4 | 15.3±4.2 ×1.5±0.5 | 10.2±1.4 |
| **F31** | purple | F | C | W to DW | 27.3±3.5 ×1.7±0.8 | 16.40±7.6 | P | 2 to 3 | 12.0±2.0 ×1.3±0.3 | 9.0±1.0 |
| **F35** | Purple | F | C | W to DW | 23.3±5.7 ×2.0±0.5 | 11.67±0.7 | S | 2 to 3 | 12.0±2.0 ×1.5±0.5 | 8.0±4.6 |
| **FUR12** | pinkish | F | C | P | 21.0±1.0 ×2.0±0.5 | 10.50±2.3 | S | 2 to 4 | 17.7±3.1 ×2.0 ±0.5 | 8.8±4.1 |
| **FUG4** | pinkish | F | C | LP | 24.7±2.5 ×1.8±0.3 | 13.45±1.1 | S | 2 | 17.0±2.0 ×1.8±0.3 | 9.3±1.9 |
| **FUG3** | yellowish | F | C | W to DW | 26.3±5.0 ×2.2±0.8 | 12.15±2.0 | S | 03 to 6 | 18.7±3.1 ×1.7±0.3 | 11.2±2.9 |
| **F57** | dark purple | F | C | LPU | 22.7±2.5 ×2.0±0.5 | 11.33±1.7 | P | 2 to 4 | 15.0±4.0 ×1.8±0.3 | 8.2±3.7 |
| **F27** | orange | A | SP | W to DW | 50.3±17.5 ×1.7±0.8 | 30.20±14.9 | S | 2 to 6 | 21.0±2.0 ×1.0±0.5 | 21.0±17.3 |
| **FUG6** | yellowish | F | C | W to DW | 37.0±4.6 ×1.7±0.8 | 22.20±7.8 | S | 2 to 3 | 26.0±2.6 ×1.5±0.5 | 17.3±5.8 |
| **F9** | purple | A | SP | W to DW | 24.0±1.0 ×1.5±0.5 | 16.00±6.0 | S | 2 to 3 | 15.0±1.0 ×0.8±0.3 | 18.0±8.7 |
| **F2** | purple | F | C | W to DW | 25.3±5.7 ×2.2±0.8 | 11.69±3.0 | S | 2 | 12.3±2.5 ×1.8±0.8 | 6.7±2.3 |
| **F18** | purple | F | C | LPU | 24.0±2.0 ×1.7±0.8 | 14.40±7.0 | P | 2 to 3 | 6.7±1.5 ×1.7±0.6 | 4.0±2.9 |
| **FUG8** | yellow | F | C | W to DW | 44.3±1.5 ×2.0±0.5 | 22.17±6.7 | S | 2 to 4 | 23.0±3.6 ×1.5±0.5 | 15.3±3.5 |
| **F47** | purple | F to LF | SP | LPU | 40.0±1.0 ×2.3±1.0 | 8.57±4.3 | S | 2 to 5 | 14.7±4.2 ×1.5±0.5 | 9.8±0.8 |
| **F16** | purple | F | C | W to DW | 26.0±1.0 ×1.7±0.8 | 15.60±7.1 | P | 2 to **7** | 11.7±2.5 ×1.7±1.0 | 7.0±13.5 |
| **F49** | purple | F | C | P | 15.3±2.5 ×1.7±0.8 | 9.20±2.9 | P | 2 to 3 | 9.7±1.5 ×2.5±0.5 | 3.9±0.2 |
| **F14** | purple | F to LF | SP | LPU | 27.0±2.0 ×2.3±1.0 | 11.57±4.2 | S | 2 to **7** | 12.7±2.1 ×2.5±0.5 | 5.1±0.4 |
| **F10** | purple | F | C | W to DW | 26.0±1.0 ×1.8±0.8 | 14.18±7.7 | P | 2 to 3 | 20.3±9.1 ×2.5±0.5 | 8.1±2.5 |
| **F58** | purple | F to LF | SP | DPU | 18.0±2.6 ×2.0±1.3 | 9.00±7.9 | B | 2 to 3 | 11.0±1.0 ×2.5±0.5 | 4.4±0.5 |
| **F32** | yellow | F to LF | SP | W to DW | 26.0±2.0 ×2.0±1.3 | 13.00±8.0 | P | 2 to 3 | 12.3±1.5 ×1.5±0.5 | 8.2±4.3 |
| **F20** | purple | F | C | W to DW | 38.0±3.0 ×1.7±0.8 | 22.80±9.3 | P | 2 to 8 | 31.3±1.5 ×1.8±0.8 | 17.1±11.2 |
| **F42** | purple | F | C | LPU | 27.0±2.0 ×1.7±0.8 | 16.20±6.7 | B | 2 to 6 | 13.0±4.6 ×1.5±06 | 8.5±7.0 |
| **F43** | yellow | F | C | W to DW | 26.0±5.6 ×2.3±1.0 | 11.14±2.6 | P | 2 to 6 | 21.3±1.5 ×2.5±0.5 | 8.5±1.6 |
| **F19** | purple | F | C | W to DW | 23.7±1.5 ×2.3±0.8 | 10.14±3.4 | P | 2 to 3 | 14.7±3.5 ×1.5±0.5 | 9.8±6.1 |
| **FUG7** | purple | A | SP | W to DW | 30.3±4.2 ×2.3±1.0 | 13.00±4.0 | P | 2 to 6 | 13.0±2.6 ×1.8 ± 0.3 | 7.1±2.2 |
| **F33** | purple | F | C | W to DW | 24.0±1.0 ×1.7±0.8 | 14.40±6.5 | P | 2 to 3 | 13.3±1.5 ×2.5±0.5 | 5.3±0.5 |
| **F48** | purple | F | C | W to DW | 13.0±2.0 ×2.0±0.5 | 6.50±1.3 | P | 2 | 5.3±1.5 ×1.5±0.5 | 3.6±0.3 |
| **F6** | white | F | C | W to DW | 37.0±3.6 ×2.3±1.0 | 15.86±5.4 | S | 2 to 5 | 12.0±2.0 ×1.5±0.5 | 8.0±1.5 |
| **F22(D2)** | purple | F | C | W to DW | 23.7±1.5 ×2.3±1.0 | 10.14±3.8 | S | 2 to **7** | 22.0±2.6 ×1.5±0.5 | 14.7±4.0 |
| **FUG5** | orange | F | C | W to DW | 24.0±1.0 ×2.3±0.8 | 10.29±3.8 | S | 2 to 3 | 15.0±5.0 ×1.5±0.5 | 9.0±1.2 |
| **F4** | purple | F to LF | SP | W to DW | 31.7±3.1 ×2.0±1.3 | 15.83±9.5 | P | 2 to 3 | 13.3±3.5 ×1.5±0.5 | 8.9±6.1 |
| **F55** | yellowish | F | C | W to DW | 25.7±1.5 ×1.5±0.5 | 17.11±5.3 | P | 2 t0 4 | 12.7±2.5 ×1.3±0.3 | 9.5±4.4 |
| **F59** | purple | F | C | LPLP | 45.0±14.7 ×2.3±1.0 | 19.29±3.8 | P | 2 to 4 | 27.0±8.5 ×2.0±0.1 | 13.3±8.0 |
| **F8** | purple | F | C | W to DW | 48.3±1.5 ×1.7±0.8 | 29.00±13.9 | S | 2 to 6 | 23.3±3.1 ×2.5±0.5 | 9.3±0.7 |
| **F26** | purple | F | C | W to DW | 0 | 0 | 0 | 0 | 13.3±3.1 ×2.5±0.5 | 5.3±0.3 |
| **FUG2** | yellowish | F | C | W to DW | 21.3±3.5 ×1.7±0.8 | 12.80±5.2 | S | 2 | 16.3±2.5 ×1.7±0.6 | 9.4±6.9 |
| **F34** | purple | F to LF | SP | W to DW | 22.0±2.0 ×2.7±0.8 | 8.25±1.6 | P | 2 to 4 | 11.7±2.5 ×1.5±0.5 | 7.8±1.0 |
| **G1-3** | pinkish | A | SP | LPLP | 31.7±2.1 ×1.5±0.5 | 21.11±6.7 | P | 2 to 3 | 22.0±5.0 ×2.5±0.5 | 8.8±2.1 |
| **Mandya 2** | dark purple | F to LF | SP | LPU | 23.7±5.5 ×2.5±0.5 | 9.47±0.4 | P | 2 to 3 | 14.3±2.1 ×2.5±0.5 | 5.7±0.7 |
| **Haveri** | light yellow | A | SP | W to DW | 51.0±4.6 ×2.3±1.0 | 21.86±7.7 | S | 2 to **7** | 30.0±5.0 ×1.8±0.3 | 16.4±1.3 |
| **Davanagere** | purple | F to LF | SP | LPU | 25.0±1.0 ×3.5±1.0 | 7.14±1.9 | B | 2 to 5 | 17.0±2.0 ×2.2±0.8 | 7.8±3.6 |
| **Mysuru** | dark purple | A | SP | PU | 24.0±1.0 ×2.3±0.8 | 10.29±3.7 | P | 2 to 3 | 16.7±3.1 ×1.5±0.5 | 11.1±6.7 |
| **B1-1** | light purple | F to LF | SP | PU | 22.3±3.1 ×2.7±1.0 | 8.38±3.1 | S | 2 to **7** | 17.0±2.0 ×1.5±0.5 | 11.3±5.9 |
| **W3-2** | dark purple | F to LF | SP | PU | 23.3±2.5 ×2.3±1.0 | 10.00±3.3 | S | 2 | 19.0±1.0 ×2.2±0.8 | 8.8±2.9 |
| **Raichur** | dark purple | F to LF | SP | LPU | 30.3±8.4 ×2.3±1.0 | 13.00±2.7 | P | 2 to 4 | 17.0±2.0 ×2.0±0.5 | 8.5±2.2 |
| **F36** | purple | F | C | W to DW | 24.7±1.5 ×2.0±0.5 | 12.33±2.5 | P | 2 to 3 | 13.0±1.0 ×2.2±0.3 | 6.0±0.9 |

Data represents mean± standard error mean (p<0.05)

**Note: SP**: Sparse, **C**: Condensed, **W to DW**: White to dirty white, **PU**: Purple, **LPU**: Light purple, **P**: Pink, **LP**: Light pink, **LPLP**: Light pink to light purple, **S**: sickle, **P**: Pointed, **B**: Blunted ends, **F to LF:** Fluffy to less fluffy, **F**: Fluffy, **A**: Appressed

**Supplementary Table 3**: Effect of *Fusarium* isolates on root length of seeds germinated in paper towel

| **Isolates** | **Root length (cm)** | **% reduction in root length** | **Virulency** |
| --- | --- | --- | --- |
| **Untreated Control** | **25** | **0** | - |
| F52, F38, Mandya, F44, Chokhla, F1, FUG, FUR12, F16, F49, F43, F19, F22(D2), Mandya2, Davengere, F8 (15) | 20-25 | 8-20 | Less |
| FUG1, F16, F44, FUG10, F43, F19, FUR12, F1, F38, Davanagere, FUG16, FUR13, F27, FUG9, F13, FUG2, F47, G1-3, F39, F34, FUR14, F8, F57, F18, F32, FUG6, F7, F3, F2, F45, F58, B1-1, F33, FUR11, F22, F28 and F25 (37) | 10-20 | 22 to 60 | Moderate |
| W3-2, F36, Raichur, Mysore, F26, F59, F55, F4, FUG5, F6, F48, FUG 7, F42, F20, F10, F14, FUG 8, F9 FUG3 FUG4, F35, F31, FUR 15, F11, F21, F12 and F46 (28) | less than 10 | 64-100 | Highly virulent |

**Supplementary Table 4:** Effect of *Fusarium* isolates on shoot length of seeds germinated in paper towel

| **Isolates** | **Shoot length (cm)** | **% Reduction in Shoot length** | **Virulency** |
| --- | --- | --- | --- |
| **Untreated control** | 45 | 0 | - |
| F52, FUR14, F19, F38, Mandya, F3, F1, F18, F22 (D2), F22 (2C), B1-1, F47, and F43 (13) | 30-43 | 4.4 to 33.3 | Less |
| F8, F34, FUG1, FUG 16, FUG 9, FUG10, FUG2, F45, FUR11, F57, G1-3, Mandya 2, Chokhla, F27, F34, F44, F16, F39, F7, F45, F58, F49, Haveri, F32, F2, W3-2, FUR 13, F32, F33, and Davanagere, (30) | 20-29.9 | 33.8-55.4 | Moderate |
| FUG2, F28, F25, F12, F21, F9, F11, FUR15, F31, F35, FUG4, FUG3, FUG8, F14, F10, F20, F42, FUG7, F48, F6, FUG5, F4, F55, F59, F26, Mysore, Raichur and F36 (28) | nil to 19.6 | 56.3-100% | Highly virulent |

**Supplementary Table 5:** Mean seedling vigour index, Mean percent disease severity and virulency behaviour of *Fusarium* spp. isolates during pathogenicity evaluation in *in-vitro, Kharif* 2020 and *Rabi* 2020-21 seasons

| **Sr. No.** | **Isolates** | **Mean Seedling Vigour index** | **Percent reduction in vigour index** | **Pathogenicity reaction** | **Isolates** | **Mean percent disease index (PDI) *Kharif* 2020** | **Pathogenicity reaction** | **Isolates** | **Mean percent disease index (PDI) *Rabi* 2020-21** | **Pathogenicity reaction** |
| --- | --- | --- | --- | --- | --- | --- | --- | --- | --- | --- |
|  | F46 | 0.0 | 100 | Virulent | FUR14 | 11.1±0.577 | Less virulent | FUG2 | 18.5±0.577 | Less virulent |
|  | F12 | 0.0 | 100 |  | FUG16 | 18.5±0.583 |  | FUG1 | 18.5±0.577 |  |
|  | F21 | 0.0 | 100 |  | FUR13 | 22.2±1.155 |  | F44 | 18.5±0.577 |  |
|  | F11 | 0.0 | 100 |  | F38 | 22.2±0.577 |  | F52 | 18.5±1.155 |  |
|  | FUR15 | 0.0 | 100 |  | F27 | 22.2±0.577 |  | FUR10 | 18.5±1.155 |  |
|  | F31 | 0.0 | 100 |  | F3 | 22.2±1.155 |  | F39 | 22.2±1.55 |  |
|  | F35 | 0.0 | 100 |  | F2 | 22.2±1.55 |  | FUR12 | 22.21.155 |  |
|  | FUG4 | 0.0 | 100 |  | F43 | 22.2±0.577 |  | Mandya2 | 22.2±0.577 |  |
|  | FUG3 | 0.0 | 100 |  | FUG7 | 22.2±1.155 |  | F19 | 25.9±0.577 | Moderately virulent |
|  | F9 | 0.0 | 100 |  | F19 | 25.9±0.583 | Moderately virulent | FUG4 | 25.9±0.577 |  |
|  | FUG8 | 0.0 | 100 |  | FUG4 | 25.9±1.155 |  | F6 | 25.9±0.577 |  |
|  | F14 | 0.0 | 100 |  | F9 | 25.9±0.577 |  | F14 | 25.9±0.577 |  |
|  | F10 | 0.0 | 100 |  | F55 | 25.9±0.621 |  | F48 | 25.9±0.577 |  |
|  | F20 | 0.0 | 100 |  | F58 | 25.9±0.577 |  | F25 | 25.9±0.577 |  |
|  | F42 | 0.0 | 100 |  | F44 | 25.9±0.577 |  | F11 | 25.9±0.577 |  |
|  | FUG7 | 0.0 | 100 |  | F16 | 25.9±0.577 |  | F3 | 25.9±0.577 |  |
|  | F48 | 0.0 | 100 |  | F47 | 29.6±1.155 |  | Haveri | 25.9±0.577 |  |
|  | F6 | 0.0 | 100 |  | FUG1 | 29.6±1.155 |  | F47 | 29.6±1.155 |  |
|  | FUG5 | 0.0 | 100 |  | F45 | 29.6±1.155 |  | F36 | 29.6±1.155 |  |
|  | F4 | 0.0 | 100 |  | FUG2 | 29.6±1.155 |  | FUG6 | 29.6±1.155 |  |
|  | F59 | 0.0 | 100 |  | F10 | 51.9±0.577 |  | F33 | 29.6±1.155 |  |
|  | F26 | 0.0 | 100 |  | F7 | 29.6±0.577 |  | F49 | 29.6±0.577 |  |
|  | Mysuru | 0.0 | 100 |  | F6 | 33.3±0.577 |  | D2 | 29.6±0.577 |  |
|  | Raichur | 0.0 | 100 |  | F31 | 33.3±0.577 |  | F32 | 29.6±0.577 |  |
|  | F36 | 0.0 | 100 |  | F36 | 33.3±0.577 |  | F9 | 33.3±0.577 |  |
|  | F55 | 10.5±5.63 | 99.5 |  | F28 | 33.3±0.577 |  | F45 | 33.3±0.577 |  |
|  | W3-2 | 588.0±5.00 | 74.9 |  | FUG5 | 33.3±0.577 |  | F10 | 33.3±0.577 |  |
|  | FUG2 | 696.4±7.43 | 70.3 |  | F34 | 33.3±0.155 |  | F27 | 33.3±0.577 |  |
|  | F33 | 732.1±7.56 | 68.8 |  | F8 | 33.3±1.155 |  | F26 | 33.3±0.577 |  |
|  | F2 | 822.2±9.12 | 65.0 |  | F22(Durgapur) | 33.3±1.155 |  | F31 | 33.3±0.577 |  |
|  | F34 | 822.6±9.94 | 64.9 | Moderately virulent | F46 | 37±1.155 |  | F8 | 33.3±0.577 |  |
|  | F25 | 822.9±13.09 | 64.9 |  | FUG6 | 37±1.155 |  | F22 | 33.3±0.577 |  |
|  | F27 | 867.6±9.37 | 63.0 |  | F12 | 37±1.155 |  | F46 | 33.3±0.577 |  |
|  | B1-1 | 872.6±9.49 | 62.8 |  | F33 | 37±1.155 |  | F20 | 33.3±0.577 |  |
|  | G1-3 | 897.1±5.87 | 61.8 |  | F14 | 37±1.155 |  | FUG8 | 33.3±0.577 |  |
|  | F18 | 907.7±9.34 | 61.3 |  | F26 | 37±0.577 |  | F42 | 33.3±0.577 |  |
|  | F22(D2) | 989.5±9.81 | 57.8 |  | F48 | 37±0.577 |  | F57 | 33.3±0.577 |  |
|  | FUR14 | 991.8±11.59 | 57.7 |  | F20 | 37±0.577 |  | FUR15 | 33.3±1.155 |  |
|  | FUG9 | 1004.4±12.00 | 57.2 |  | F4 | 40.7±1.155 |  | F21 | 33.3±1.155 |  |
|  | F47 | 1005.4±8.56 | 57.2 |  | F11 | 40.7±0.577 |  | F35 | 33.3±1.155 |  |
|  | Mandya 2 | 1016.9±11.02 | 56.7 |  | F49 | 40.7±0.589 |  | Mysuru | 33.3±1.155 |  |
|  | F52 | 1024.9±10.44 | 56.3 |  | FUG8 | 44.4±0.577 |  | W3-2 | 33.3±1.155 |  |
|  | F16 | 1057.4±11.55 | 54.9 |  | F42 | 44.4±0.577 |  | Mandya | 33.3±1.155 |  |
|  | F22 (2C) | 1060.1±14.13 | 54.8 |  | D2 | 44.4±0.577 |  | F58 | 37.0±0.577 |  |
|  | FUG10 | 1081.5±20.32 | 53.9 |  | FUG3 | 48.1±1.155 |  | F16 | 37±0.577 |  |
|  | F43 | 1090.7±10.08 | 53.5 |  | F52 | 48.1±1.155 |  | F34 | 37±0.577 |  |
|  | F7 | 1158.3±13.00 | 33.6 |  | F57 | 48.1±0.577 |  | F59 | 37±0.577 |  |
|  | F8 | 1219.4±10.35 | 48.0 |  | FUR10 | 48.1±1.155 |  | F18 | 37±1.155 |  |
|  | F57 | 1226.0±6.64 | 47.8 |  | F21 | 51.9±0.577 | Virulent | G1-3 | 37.0±155 |  |
|  | F45 | 1246.4±11.12 | 46.9 |  | F18 | 51.9±0.577 |  | FUR11 | 40.7±0.577 |  |
|  | F28 | 1253.2±12.35 | 46.6 |  | FUR15 | 51.9±0.577 |  | B1-1 | 40.7±1.155 |  |
|  | FUR13 | 1270.7±10.71 | 45.9 |  | F13 | 51.9±0.577 |  | FUG5 | 44.4±1.153 |  |
|  | FUR12 | 1282.8±15.40 | 45.3 |  | F1 | 55.6±0.577 |  | FUG9 | 44.4±0.566 |  |
|  | Chokhla | 1319.8±15.62 | 43.8 |  | F25 | 55.6±0.577 |  | F55 | 48.1±0.575 |  |
|  | FUG6 | 1352.1±8.86 | 42.4 |  | FUR12 | 55.6±1.155 |  | F28 | 48.1±0.579 |  |
|  | Mandya | 1407.4±16.30 | 40.0 |  | F59 | 59.3±1.155 |  | F13 | 51.9±0.577 | Virulent |
|  | F1 | 1419.6±15.49 | 39.5 |  | Chokhla | 59.3±1.155 |  | F1 | 51.9±1.155 |  |
|  | FUR11 | 1432.4±10.13 | 39.0 |  | F32 | 59.3±0.577 |  | Davangere | 51.9±0.577 |  |
|  | F38 | 1463.9±9.53 | 37.6 |  | FUR11 | 63.0±0.577 |  | Chokhla | 55.6±1.155 |  |
|  | F44 | 1502.3±15.04 | 36.0 | Less virulent | F35 | 66.7±1.155 |  | Raichur | 66.6±0.024 |  |
|  | F7 | 1558.1±13.00 | 33.6 |  |  |  |  |  |  |  |
|  | F3 | 1561.1±13.68 | 33.5 |  |  |  |  |  |  |  |
|  | FUG16 | 1570.8±11.64 | 33.1 |  |  |  |  |  |  |  |
|  | F39 | 1572.4±8.13 | 33.0 |  |  |  |  |  |  |  |
|  | Davangere | 1574.5±7.53 | 32.9 |  |  |  |  |  |  |  |
|  | F13 | 1613.3±13.87 | 31.3 |  |  |  |  |  |  |  |
|  | F49 | 1619.6±13.05 | 31.0 |  |  |  |  |  |  |  |
|  | F32 | 1662.3±12.34 | 29.2 |  |  |  |  |  |  |  |
|  | FUG1 | 1807.6±12.49 | 23.0 |  |  |  |  |  |  |  |
|  | F58 | 1826.0±7.75 | 22.2 |  |  |  |  |  |  |  |
|  | F19 | 2246.5±7.90 | 4.3 |  |  |  |  |  |  |  |
|  | control | 2347.0±10.86 | 0.0 |  |  |  |  |  |  |  |

Data based on mean of three replications. Pairwise mean comparison of isolates was done using Tukey’s HSD test at *P*=0.05. Data represents mean± standard error mean
